# Designing broadband pulsed dynamic nuclear polarization sequences in static solids

**DOI:** 10.1126/sciadv.abq0536

**Published:** 2022-07-15

**Authors:** Nino Wili, Anders Bodholt Nielsen, Laura Alicia Völker, Lukas Schreder, Niels Chr. Nielsen, Gunnar Jeschke, Kong Ooi Tan

**Affiliations:** ^1^ Department of Chemistry and Applied Biosciences, Laboratory of Physical Chemistry, ETH Zurich, Vladimir-Prelog-Weg 2, 8093 Zurich, Switzerland.; ^2^ Interdisciplinary Nanoscience Center (iNANO) and Department of Chemistry, Aarhus University, Gustav Wieds Vej 14, DK-8000 Aarhus C, Denmark.; ^3^ Laboratoire des Biomolécules, LBM, Département de Chimie, École Normale Supérieure, PSL University, Sorbonne Université, CNRS, 75005 Paris, France.

## Abstract

Dynamic nuclear polarization (DNP) is a nuclear magnetic resonance (NMR) hyperpolarization technique that mediates polarization transfer from unpaired electrons with large thermal polarization to NMR-active nuclei via microwave (mw) irradiation. The ability to generate arbitrarily shaped mw pulses using arbitrary waveform generators allows for remarkable improvement of the robustness and versatility of DNP. We present here novel design principles based on single-spin vector effective Hamiltonian theory to develop new broadband DNP pulse sequences, namely, an adiabatic version of XiX (X–inverse X)–DNP and a broadband excitation by amplitude modulation (BEAM)–DNP experiment. We demonstrate that the adiabatic BEAM-DNP pulse sequence may achieve a ^1^H enhancement factor of ∼360, which is better than ramped-amplitude NOVEL (nuclear spin orientation via electron spin locking) at ∼0.35 T and 80 K in static solids doped with trityl radicals. In addition, the bandwidth of the BEAM-DNP experiments (~50 MHz) is about three times the ^1^H Larmor frequency. The generality of our theoretical approach will be helpful in the development of new pulsed DNP sequences.

## Teaser

A powerful generalized theoretical framework to design efficient broadband pulsed DNP sequences at low fields is presented.

## INTRODUCTION

Dynamic nuclear polarization (DNP) is a powerful tool to increase the sensitivity of nuclear magnetic resonance (NMR) by transferring the much higher polarization of electron spins to nuclear spins with a theoretical maximum enhancement factor ε ∼ 658 (*1*, *2*) for ^1^H. The hyperpolarization method allows one to study systems that suffer from poor NMR sensitivity with reduced measurement time or cost. For instance, a DNP experiment with ε ∼ 100 performed in 1 hour would have taken ∼1 year without microwave (mw) irradiation for the same-quality spectrum. This opens up the possibility to extract important structural information from small molecules, biological samples, or inorganic materials that are otherwise inaccessible because of poor NMR sensitivity.

Since the discovery of DNP in the 1950s (*3*), tremendous progress has been made, and there are two main DNP methods: dissolution DNP (*4*) and in situ magic-angle spinning (MAS) solid-state DNP NMR (*5*). For the former category, static samples are typically polarized at low temperatures (<2 K) and moderate magnetic fields (3.4 to 10.1 T) (*6*), where the electron polarization approaches unity. Following that, the sample undergoes a dissolution process before it is transported to a high-resolution NMR magnet or MRI system for detection in solution state. The in situ static sample or MAS DNP NMR approach performs the hyperpolarization and NMR detection process on solid samples in the same high-resolution magnet typically at temperatures below 100 K. These contemporary DNP approaches use continuous-wave (CW) mw irradiation, where the amplitude, phase, and frequency are not modulated at all, or only slowly. Hence, only four main CW-DNP mechanisms have been discovered so far, namely, the Overhauser effect (OE) (*3*), the solid effect (SE) (*7*, *8*), the cross effect (CE) (*9*, *10*), and thermal mixing (TM) (*11*, *12*). In comparison, hundreds of NMR pulse sequences have been invented to date, for purposes ranging from polarization transfer, distance measurement, to determination of dynamics and chemical environments, etc. Even in electron paramagnetic resonance (EPR) experiments at low fields, where high-power mw sources are available, there is only one experiment that uses DNP as a polarization transfer step (*13*) to detect hyperfine-coupled nuclei. Although pulsed mw irradiation is expected to improve DNP performances (*14*), the effect of modulated pulsed irradiation can be complicated, and a generalized theoretical approach is required to design the right pulse sequences. An important aspect in pulse sequence design is the excitation bandwidth. The EPR linewidths of many radicals are often broad, especially at high fields due to large g-anisotropy. For instance, the linewidth of a typical nitroxide radical is ∼1 GHz at 9.4 T (*15*), which is orders of magnitude higher than the electron Rabi fields conferred by the currently available mw power. The aim of this study is to demonstrate—albeit at lower field—ways to design broadband and efficient DNP techniques exploiting shaped pulses generated by arbitrary waveform generators (AWGs) using different mw powers.

**Fig. 1.**
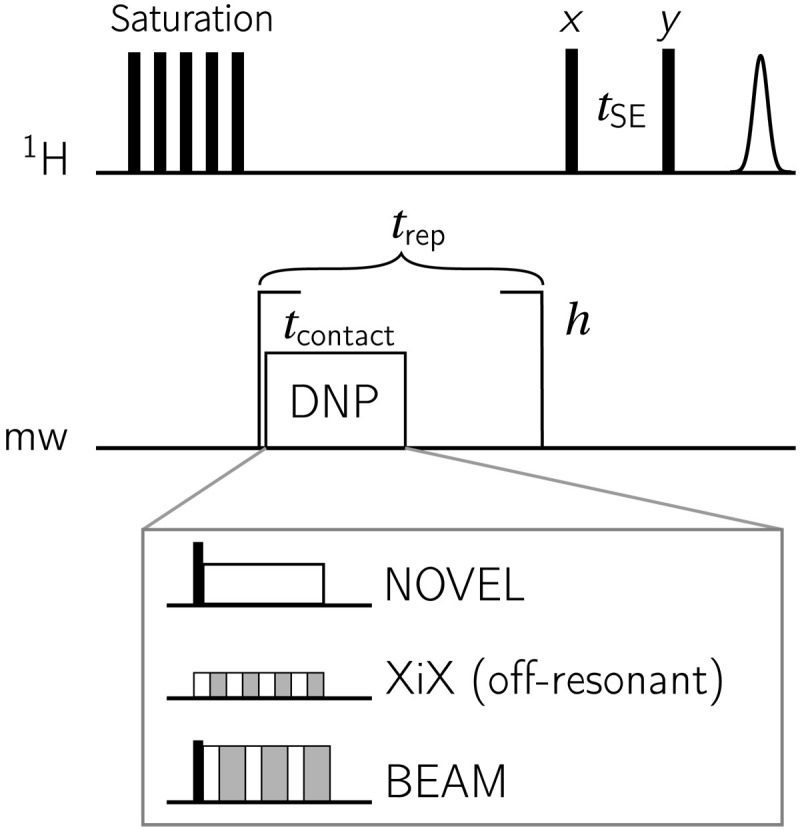
Schematic diagram of various pulsed DNP sequences (NOVEL, XiX, and BEAM) repeated by *h* loops before ^1^H-NMR detection with a solid echo.

Several pulsed DNP techniques have been described earlier (*16*–*27*), where nuclear spin orientation via electron spin locking (NOVEL) (*16*–*19*) is the DNP analog of cross-polarization (CP) except that there is no radio-frequency (rf) irradiation on the nuclei. We would like to emphasize that the design principle of the time-optimized pulsed DNP (TOP-DNP) sequence (*27*) is substantially different from others; i.e., the initially truncated electron–nuclear dipolar couplings by the nuclear Zeeman term are strategically reintroduced by pulsed mw irradiation to mediate DNP. The way that TOP-DNP reintroduces these couplings is mathematically similar to and inspired by the dipolar recoupling techniques in MAS solid-state NMR spectroscopy, where rf irradiation interferes with the rotational averaging of dipolar couplings. Following this, a phase-alternating low-power X–inverse X (XiX)–DNP sequence (Fig. 1) has been designed recently using the operator-based Floquet theory (*28*).

In this work, we introduce a design strategy based on single-spin vector effective Hamiltonian theory (*29*, *30*), which incorporates Fourier coefficients [exploited in the operator-based Floquet theory (*31*)] into average Hamiltonian theory (AHT) and—more importantly—is applicable to sequences that lead to an arbitrary overall rotation of the spins. This theoretical framework is applicable to any arbitrary periodic mw irradiation scheme and will be used here for designing broadband DNP experiments on static samples. We exemplify this for two simple sequences. First, we make the previously introduced XiX-DNP adiabatic by modulating the pulse lengths. Second, we increase the bandwidth of NOVEL using an amplitude-modulation (or phase-inversion) scheme, yielding the BEAM sequence shown in Fig. 1. We will examine these sequences by numerical simulations and experiments at 0.35 T/9.8 GHz/15 MHz on OX063 trityl radicals doped in a glycerol-water mixture at 80 K.

## THEORY

### Overview

An efficient DNP experiment requires a fast transfer of a large fraction of thermal electron spin polarization to the nuclear spins. By applying time-dependent instead of continuous mw irradiation, pulsed DNP provides a much larger parameter space for optimizing this transfer. On the downside, this approach generates a more complicated time dependence of the spin Hamiltonian. This time dependence carries over to the equation of motion for the spin states, preventing intuitive understanding of the polarization transfer. While the simple time dependence of cw irradiation can be removed in a simple rotating-wave approximation, removal of the time dependence for pulsed DNP requires more sophisticated approaches, such as AHT or Floquet theory. Here, we introduce an approach that directly provides the DNP matching conditions and the effective Hamiltonians, which contain scaling factors that dictate the DNP performances. To achieve this, we subject the overall spin Hamiltonian to a series of transformations that lead to a convergent effective Hamiltonian. By expanding this Hamiltonian as a Fourier series, we can easily characterize the resonance conditions and the effective couplings. This approach is inspired by the single-vector effective Hamiltonian approaches introduced recently for the description of solid-state NMR dipolar recoupling (*29*) and liquid-state NMR isotropic mixing (*30*).

We show in the Detailed theory section below that the effective Hamiltonian of an electron–nuclear two-spin system (*S* and *I*, respectively) subject to a periodic mw irradiation with modulation frequency ω_m_ can be written as

$$\begin{array}{c} H_{\text{eff}}=\frac{Ba_{\mp }}{4}({\tilde{S}}^{-}I^{\pm }+{\tilde{S}}^{+}I^{\mp })\\ -\omega_{\text{eff}}^{\left(S\right)}{\tilde{S}}_{z}+\omega_{\text{eff}}^{\left(I\right)}I_{z} \end{array}$$ (1)

where *B* is the pseudo-secular hyperfine coupling, which originates from the electron–nuclear dipolar coupling and averages to zero in solution. The scaling factor *a*_∓_ depends only on the pulse sequence and determines the DNP transfer efficiency. It can be positive or negative, because both a zero-quantum (ZQ) and double-quantum (DQ) transfer is possible, leading to opposite signs of the nuclear polarization. The effective field of the electron spin is denoted by $\omega_{\text{eff}}^{\left(S\right)}{\tilde{S}}_{z}$ and describes the overall rotation of the electron spin over one basic element of the periodic pulse sequence. In the case of CW irradiation, the effective field is simply the vector sum of the Rabi field in the transverse plane and offset frequency along *z*, but it can be generalized to arbitrary periodic sequences. Note that the direction of the effective field does not usually coincide with the usual *z* axis of the rotating frame. This is important, because only the projection of electron polarization along the effective field is transferred to the nuclei. The analogous quantity of the nuclear spin is given by $\omega_{\text{eff}}^{\left(I\right)}I_{z}$. In the absence of rf irradiation, it only depends on the nuclear Zeeman frequency ω*_I_* and the modulation frequency ω_m_

$$\omega_{\text{eff}}^{\left(I\right)}=\omega_{I}-k_{I}\omega_{m}\;\text{with}\;k_{I}=\text{round}(\frac{\omega_{I}}{\omega_{m}})$$ (2)

Note that AHT usually only deals with sequences where the spins end up at their starting position after a small number of cycles, and our approach does not have such a limitation. The form of the effective Hamiltonian is well known in NMR and appears in virtually every discussion of heteronuclear polarization transfer, most notably CP. It is thus not unusual that many sequences and concepts from (solid-state) NMR can be adapted for DNP. For example, NOVEL is the DNP analog of CP, and the idea of ramped-amplitude (RA) NOVEL is similar to RA CP. The notable major differences between the two magnetic resonance cases are the magnitudes of the couplings and the presence of a significant pseudo-secular coupling in DNP.

To design an efficient polarization transfer experiment, we need to fulfill three criteria, (i) the scaling factor *a*_∓_ should be large, (ii) the initial density operator ρ_0_ should be aligned with the electron effective field, and (iii) the mismatch of the effective fields should be minimal. To address the first two points, we introduce the transfer parameter *f*_∓_

$$f_{\mp }=\langle \rho_{0}|{\tilde{S}}_{z}\rangle a_{\mp }$$ (3)

For point (iii), we express the mismatch as

$$\Delta \omega_{\text{eff}}^{\mp }=\omega_{\text{eff}}^{\left(S\right)}\pm \omega_{\text{eff}}^{\left(I\right)}$$ (4)

A maximal transfer can be achieved when the resonance condition is exactly fulfilled $\Delta \omega_{\text{eff}}^{\mp }=0$. While it is easy to satisfy the resonance condition for a single spin packet, it becomes more challenging when multiple spin packets with different offset frequencies Ω*_S_* are present. In addition, the presence of mw power inhomogeneity will result in distributions of the electron nutation frequency ω_1_ across the sample. The electron effective field $\omega_{\text{eff}}^{\left(S\right)}(\Omega_{S},\omega_{1})$ and, hence, the mismatch are governed by these two parameters

$$\Delta \omega_{\text{eff}}^{\mp }=\Delta \omega_{\text{eff}}^{\mp }(\Omega_{S},\omega_{1})$$ (5)

Accordingly, sequence design aims at maximizing the scaling factor while minimizing the mismatch. To determine the mismatches (Eq. 5), one needs to calculate the electron effective field for all possible Ω*_S_* and ω_1_ values. The calculation of the effective field is particularly fast, because it only involves classical three-dimensional rotations. If the effective field and, subsequently, the resonance mismatch are calculated for a set of electron offsets, the bandwidth of a sequence can be determined quite easily. Under the specific conditions outlined in the Detailed theory section, our approach thus reduces a two-spin problem to a single-spin problem, which reduces the complexity and facilitates design. In this work, we provide a simple program that calculates the scaling factors and effective fields from a given irradiation scheme (including an explicit offset Ω*_S_*). This allows for easy evaluation of resonance conditions and a direct quantification of the bandwidth. For the particular sequences studied in this work, it is possible to express the scaling factors and effective fields analytically in some limiting cases. However, the numerical program can treat arbitrary cases where it will be tedious or impossible in other known approaches.

### Adiabatic sweeps

So far, we assumed that the basic pulse sequence element is repeated periodically with the same parameters. However, it is known in NMR that an adiabatic sweep through the resonance condition can significantly improve the sequence robustness toward offsets and inhomogeneities. Note that the process not only needs to be adiabatic for the electron spin, i.e., it needs to follow the effective field, but the process also has to correspond to an adiabatic inversion in the ZQ subspace (or DQ, depending on the resonance condition). To achieve this, the mismatch of the effective fields has to be swept (see below for detailed mathematical description) from large positive, through zero, then to large negative values (or vice versa). In simple terms, the *z* operator in the ZQ subspace corresponds to the difference of electron and nuclear polarization, and the inversion of this operator corresponds to an adiabatic electron–nuclear polarization transfer.

**Fig. 2.**
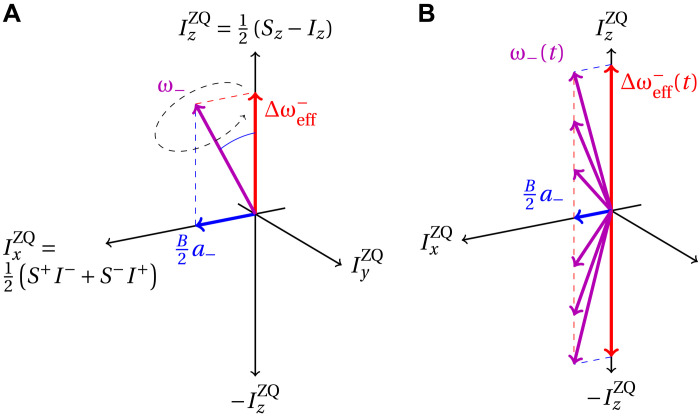
Diagram of the effective (fictitious) spin-1/2 subspaces involved in DNP in the ZQ subspace. (**A**) Diabatic (“sudden”) sequence with a constant mismatch. (**B**) Adiabatic sequence with slowly changing mismatch of the nuclear and electron effective fields. The effective “mismatch” field is slowly dragged from ***+****z* to −*z*, corresponding to full polarization transfer. Note that *a*_∓_ can be slowly time dependent, too. For simplicity, terms proportional to $I_{y}^{\text{ZQ}}$ are ignored in this illustration.

Figure 2A shows the ZQ subspace for time-independent effective fields. The mismatch (red) corresponds to a *z* offset in this subspace, while the effective coupling (blue) corresponds to a transverse field. If the mismatch is nonzero, the effective field in the ZQ subspace (purple) is not in the transverse plane. Thus, the *z* component of the ZQ subspace can never be fully inverted (illustrated by the black dashed cone) and only a small amount of polarization is transferred. On the other hand, Fig. 2B shows the case where the mismatch is slowly swept through zero, which adiabatically inverts the *z* component and leads to a full polarization transfer. This does not strongly depend on the value of the mismatch at the start and end of the sweep; i.e., this is where the robustness of many adiabatic sequences originates from.

We want to emphasize that the electron effective fields, $\omega_{\text{eff}}^{\left(S\right)}$, and, thus, the mismatch can be expressed for any periodic sequence. This implies that one can sweep many parameters including the offset, amplitude, phase, and pulse lengths to implement an adiabatic sweep. In this work, we modulate the pulse lengths in a two-pulse sequence to achieve an adiabatic transfer.

## Detailed theory

### Deriving the effective Hamiltonian

In this section, we give a detailed derivation of the effective Hamiltonian in Eq. 1. This will allow interested readers to follow exactly how the scaling factors are calculated and use the provided MATLAB scripts to develop their own DNP sequences.

The theory outlined in the following is extendable to systems with multiple electrons and nuclei. For simplicity, we stick to a two-spin system composed of one electron spin (*S*) and one nuclear spin (*I*). The laboratory-frame Hamiltonian is given by

$$H=\omega_{S}S_{z}+H_{\text{mw}}+\vec{S}\cdot A\cdot \vec{I}+\omega_{I}I_{z}$$ (6)

with ω*_S_* = −γ_e_*B*_0_ and ω*_I_* = −γ_n_*B*_0_ being angular frequencies for the electron and nuclear Zeeman interactions, respectively (for an e-^1^H system ω*_S_* > 0 and ω*_I_* < 0). γ, **A**, H_mw_, and *B*_0_ refer to the gyromagnetic ratio, the hyperfine coupling tensor, the Hamiltonian of the mw irradiation, and the external static magnetic field along the *z* axis, respectively. We first transform to the electron rotating frame, thus removing the fast time dependence at the mw frequency within the rotating-wave approximation. We further use the high-field approximation with respect to the electron spin to obtain the first-order effective Hamiltonian

$$\overset{-}{\tilde{H}}=\underset{H_{\text{control}}}{\underset{︸}{\Omega_{S}S_{z}+{\tilde{H}}_{\text{mw}}}}+\underset{H^{\prime }}{\underset{︸}{A_{\mathrm{zz}}S_{z}I_{z}+BS_{z}I_{x}+\omega_{I}I_{z}}}$$ (7)

where Ω*_S_* = ω*_S_* − ω_mw_ is the mw offset frequency; *A_zz_* and $B=\sqrt{A_{\mathrm{zx}}^{2}+A_{\mathrm{zy}}^{2}}$ are the secular and pseudosecular coupling, respectively. Note that the *B* term originates purely from the dipolar coupling, which is averaged to zero in solution state. We will restrict our discussions to spin systems in the weak coupling regime where ∣*A_zz_*∣,∣*B*∣ ≪ ∣ω*_I_*∣.

At this point, ${\tilde{H}}_{\text{mw}}$ still features the slower time dependence arising from amplitude, phase, and frequency modulation of the mw irradiation. We remove this time dependence by transformation into the interaction frame with the control field H_control_, which contains information on the amplitude- and frequency-modulation function of an irradiation waveform

$$\begin{array}{cc} \tilde{H\prime } & =U_{\text{control}}^{†}\;H\prime \;U_{\text{control}}\\ & =\sum_{\chi =x,y,z}R_{\chi z}^{\left(\text{control}\right)}(t)S_{\chi }(A_{\mathrm{zz}}I_{z}+BI_{x})+\omega_{I}I_{z} \end{array}$$ (8)

where $U_{\text{control}}(t)=\hat{T}\text{exp}\;(-i\int_{0}^{t}H_{\text{control}}(\tau )d\tau )$ and $\hat{T}$ is the Dyson time-ordering operator. The time-dependent rotation matrix $R_{\chi z}^{\left(\text{control}\right)}$ represents the interaction-frame trajectory of the electron spin under the irradiation waveform. For the special case of a pulse sequence, the waveform features jumps in phase, amplitude, or frequency. The effective time-independent Hamiltonian can be computed using standard average Hamiltonian theory, provided H_control_(*t*) is periodic over a given period τ_m_ = 2π/ω_m_ (where ω_m_ is the modulation frequency of the pulse sequence), i.e., H_control_(*t*) = H_control_(*t* + τ_m_). We note that a periodic control Hamiltonian does not necessarily lead to a periodic time dependence of the spin states. In mathematical terms, this means that the control propagator *U*_control_ over one cycle, which is equivalent to $R_{\chi z}^{\left(\text{control}\right)}$, is not necessarily an identity operator.

Intuitive understanding of the polarization transfer is simplest in a periodicity-adapted frame where the control propagator is an identity operator, as in this case, time dependence can be completely removed by applying AHT, as we shall demonstrate below. The *z* axis of this frame is aligned with the effective field (ω_eff_) (*29*, *30*), whose magnitude and direction can be determined using quaternion algebra (*32*–*34*). We indicate the periodicity-adapted frame in the following with a tilde on the $\tilde{S}$-spin operators. This leads to

$$\tilde{H\prime }=\sum_{\chi =x,y,z}R_{\chi z}^{\left(\text{eff}\right)}(t){\tilde{S}}_{\chi }(A_{\mathrm{zz}}I_{z}+BI_{x})-\omega_{\text{eff}}^{\left(S\right)}{\tilde{S}}_{z}+\omega_{I}I_{z}$$ (9)

with

$$\begin{array}{cc} R_{\chi z}^{\left(\text{eff}\right)}(t) & ={\left[R_{z}(-\omega_{\text{eff}}^{\left(S\right)}t)\cdot R^{\left(\text{flip}\right)}(\beta )\cdot R^{\left(\text{control}\right)}\right]}_{\chi z}(t)\\ & =\sum_{k=-\infty }^{\infty }a_{\chi z}^{\left(k\right)}e^{\mathrm{ik}\omega_{m}t} \end{array}$$ (10)

At this point, we have found a three-step transformation that leads to a time-independent description for irradiation that lasts over a large number of periods. This transformation is composed of (i) going into the control frame, (ii) flipping the coordinate system by an angle β so that it is aligned with $\omega_{\text{eff}}^{\left(S\right)}{\tilde{S}}_{z}$, and (iii) rotating the frame by an angle $\omega_{\text{eff}}^{\left(S\right)}t$ around the new *z* axis (*29*, *30*). The time-dependent rotation matrix $R_{\chi z}^{\left(\text{eff}\right)}(t)$ defined by Eq. 10 has the desired property of being cyclic, i.e., ${\left[R_{\chi z}^{\left(\text{eff}\right)}(t+\tau_{m})\right]}_{\chi z}=R_{\chi z}^{\left(\text{eff}\right)}(t)$. The program in the Supplementary Materials performs the above transformation numerically and subsequently calculates the Fourier coefficients $a_{\chi z}^{\left(k\right)}$. More details can be found in the Supplementary Materials. Up to periodicity, a time-independent description is achieved in the second line of Eq. 10 by expansion into a Fourier series, which provides the coefficients $a_{\chi z}^{\left(k\right)}$ as performance parameters of the irradiation waveform. The term $-\omega_{\text{eff}}^{\left(S\right)}{\tilde{S}}_{z}$ incorporates the Coriolis term originating from step (iii) above.

While the periodicity-adapted frame provides a simple and easy-to-average description of the time dependence of electron spin irradiation, it is still complicated to see how polarization builds up on the nuclear spin. We solve this problem by transforming the nuclear part (*I*) of the Hamiltonian into a periodicity-matching frame by finding the multiple *k_I_* of the nuclear Larmor frequency ω*_I_* that most closely matches the mw modulation frequency ω_m_. The remaining mismatch is the *I*-spin effective field

$$\omega_{\text{eff}}^{\left(I\right)}=\omega_{I}-k_{I}\omega_{m}\;\text{with}\;k_{I}=\text{round}(\frac{\omega_{I}}{\omega_{m}})$$ (11)

which is conceptually similar to a resonance offset. Transformation into the periodicity-matching frame is achieved by a propagator *U*_eff_ = exp (−*ik_I_*ω_m_*tI_z_*), yielding

$$\begin{array}{c} \tilde{\tilde{H}}\prime =U_{\text{eff}}^{†}\;\tilde{H\prime }\;U_{\text{eff}}-k_{I}\omega_{m}I_{z}=\sum_{\chi =x,y,z}\sum_{k=-\infty }^{\infty }a_{\chi z}^{\left(k\right)}e^{\mathrm{ik}\omega_{m}t}{\tilde{S}}_{\chi }\\ \times (A_{\mathrm{zz}}I_{z}+\frac{B}{2}(e^{ik_{I}\omega_{m}t}I^{+}+e^{-ik_{I}\omega_{m}t}I^{-}))-\omega_{\text{eff}}^{\left(S\right)}{\tilde{S}}_{z}+\omega_{\text{eff}}^{\left(I\right)}I_{z} \end{array}$$ (12)

Last, we remove the remaining periodic time dependence by first-order AHT. In this step, all terms of the sum over *k* vanish, except for the ones with *k* being either = ±*k_I_* or =0. The time-independent Hamiltonian is

$$\begin{array}{c} {\bar{\tilde{\tilde{H}}}}^{\left(1\right)}=A_{\mathrm{zz}}\sum_{\chi =x,y,z}a_{\chi z}^{\left(0\right)}{\tilde{S}}_{\chi }I_{z}+\frac{B}{2}{\tilde{S}}_{z}(a_{\mathrm{zz}}^{\left(-k_{I}\right)}I^{+}+a_{\mathrm{zz}}^{\left(k_{I}\right)}I^{-})\\ +\frac{B}{4}(a_{+z}^{\left(-k_{I}\right)}{\tilde{S}}^{+}I^{+}+a_{-z}^{\left(-k_{I}\right)}{\tilde{S}}^{-}I^{+}+a_{+z}^{\left(k_{I}\right)}{\tilde{S}}^{+}I^{-}+a_{-z}^{\left(k_{I}\right)}{\tilde{S}}^{-}I^{-})-\omega_{\text{eff}}^{\left(S\right)}{\tilde{S}}_{z}+\omega_{\text{eff}}^{\left(I\right)}I_{z} \end{array}$$ (13)

where we have defined $a_{\mathrm{xz}}^{\left(q\right)}{\tilde{S}}_{x}+a_{\mathrm{yz}}^{\left(q\right)}{\tilde{S}}_{y}=\frac{1}{2}(a_{+z}^{\left(q\right)}{\tilde{S}}^{+}+a_{-z}^{\left(q\right)}{\tilde{S}}^{-})$ and $a_{\pm z}^{\left(q\right)}=a_{\mathrm{xz}}^{\left(q\right)}\mp ia_{\mathrm{yz}}^{\left(q\right)}$. Since any spin Hamiltonian is Hermitian, we must have $a_{-z}^{\left(q\right)}={\left(a_{+z}^{\left(-q\right)}\right)}^{*}$. Representation of the transfer terms by ladder operators provides an intuitive classification of the transfer pathways.

### Identifying resonance conditions and scaling factors

We note that the form of the effective Hamiltonian ${\bar{\tilde{\tilde{H}}}}^{\left(1\right)}$ (Eq. 13) applies to any periodic mw waveform acting on a two-spin electron-nucleus system in the regime where ∣*A_zz_*∣,∣*B*∣ ≪ ∣ω*_I_*∣. The details of the waveform (or pulse sequences) are encoded in the scaling factors *a*_χ*z*_ that are specific to a given resonance condition. We expect that differences in transfer efficiency between waveforms and between different resonance conditions for the same waveform are related to these scaling factors. This relation is discussed in the following.

In polarization-transfer experiments, contributions by the zero- or double-quantum (ZQ or DQ) operators compensate each other. Hence, it is necessary to select only one of these types and to suppress all other noncommuting operators. This is achieved by matching the effective fields ($-\omega_{\text{eff}}^{\left(S\right)}{\tilde{S}}_{z},\omega_{\text{eff}}^{\left(I\right)}I_{z}$) in Eq. 13. At the resonance condition $\omega_{\text{eff}}^{\left(S\right)}=-\omega_{\text{eff}}^{\left(I\right)}$, only the ZQ operators (${\tilde{S}}^{\pm }I^{\mp }$) contribute, because the DQ terms (${\tilde{S}}^{\pm }I^{\pm }$) are truncated by the larger noncommuting $I_{z}+{\tilde{S}}_{z}$ term. Likewise, for $\omega_{\text{eff}}^{\left(S\right)}=\omega_{\text{eff}}^{\left(I\right)}$, only the DQ terms remain due to analogous truncation in the ZQ subspace. All other terms can be neglected as long as they are much smaller than the effective fields. This is a good approximation for weakly coupled protons involved in DNP and was checked by numerical simulations. The *A_zz_$\tilde{S}$_z_I_z_* term commutes with the effective fields and is thus not truncated. It is, however, inconsequential, as it shifts both energy levels in the same direction within the respective subspace (ZQ or DQ). Thus, the energy difference and resonance conditions remain unchanged. By neglecting the terms discussed above, we obtain a simplified Hamiltonian for the DNP ZQ and DQ resonance conditions, ($\omega_{\text{eff}}^{\left(S\right)}\approx \mp \omega_{\text{eff}}^{\left(I\right)}$), respectively, and we arrive at the Hamiltonian discussed in Eq. 1

$$\begin{array}{cc} {\overset{-}{\tilde{\tilde{H}}}}_{\text{ZQ},\text{DQ}}^{\left(1\right)} & =H_{\text{eff}}\\ & =\frac{B}{4}(a_{-z}^{\left(\mp k_{I}\right)}{\tilde{S}}^{-}I^{\pm }+a_{+z}^{\left(\pm k_{I}\right)}{\tilde{S}}^{+}I^{\mp }) \end{array}$$ (14)

$$-\omega_{\text{eff}}^{\left(S\right)}{\tilde{S}}_{z}+\omega_{\text{eff}}^{\left(I\right)}I_{z}$$ (15)

Thus, the desired transfer of electron spin polarization, proportional to the expectation value of ${\tilde{S}}_{z}$ for the initial density operator ρ_0_, to *I_z_* can be calculated by using $U=\text{exp}\;(-i{\bar{\tilde{\tilde{H}}}}_{\text{ZQ},\text{DQ}}^{\left(1\right)}t):$

$$\begin{array}{cc} \langle I_{z}\rangle (t) & =\frac{\gamma_{e}}{\gamma_{n}}\langle \rho_{0}|{\tilde{S}}_{z}\rangle \mathrm{Tr}\{U{\tilde{S}}_{z}U^{†}I_{z}\}/\mathrm{Tr}\{I_{z}^{2}\}\\ & =\pm \frac{\gamma_{e}}{\gamma_{n}}\langle \rho_{0}|{\tilde{S}}_{z}\rangle \frac{B^{2}a_{\mp }^{2}}{4\omega_{\mp }^{2}}\overset{2}{\text{sin}}(\frac{1}{2}\omega_{\mp }t) \end{array}$$ (16)

Equation 16 provides two DNP performance parameters. The unitless scaling factor

$$a_{\mp }=\sqrt{a_{-z}^{\left(\mp k_{I}\right)}a_{+z}^{\left(\pm k_{I}\right)}}$$ (17)

governs the transfer efficiency, while the DNP buildup frequency

$$\omega_{\mp }=\sqrt{B^{2}a_{\mp }^{2}/4+{\left(\Delta \omega_{\text{eff}}^{\mp }\right)}^{2}}$$ (18)

increases with increasing “mismatch” of the effective fields

$$\Delta \omega_{\text{eff}}^{\mp }(\Omega_{S})=\omega_{\text{eff}}^{\left(S\right)}(\Omega_{S})\pm \omega_{\text{eff}}^{\left(I\right)}$$ (19)

Maximum transfer is attained at the matching condition $\Delta \omega_{\text{eff}}^{\mp }(\Omega_{S})=0$, where Eq. 16 simplifies to

$${\langle I_{z}\rangle }_{\text{matched}}(t)=\pm \frac{\gamma_{e}}{\gamma_{n}}\langle \rho_{0}|{\tilde{S}}_{z}\rangle {\text{sin}}^{2}(\frac{1}{4}Ba_{\mp }t)$$ (20)

and only the buildup rate, but not the maximum nuclear polarization, depends on the scaling coefficient *a*_∓_. Note that the mismatch depends on, but is not the same as the electron offset Ω*_S_*. While the buildup is faster in case of a larger mismatch, the transfer amplitude is lower, similar to the situation of an off-resonance pulse in a two-level system. The prefactor $\frac{\gamma_{e}}{\gamma_{n}}{\langle \rho }_{0}|{\tilde{S}}_{z}\rangle$ in Eq. 20 highlights that only the part of the electron density operator projected onto the effective field will be transferred to the nucleus. Therefore, we can quantify efficiency of an irradiation scheme at the matching condition semiquantitatively by the transfer parameter

$$f_{\mp }=\langle \rho_{0}|{\tilde{S}}_{z}\rangle a_{\mp }$$ (21)

Note that the choice of the effective field is not unique. With the convention $|\omega_{\text{eff}}^{\left(S\right)}|,|\omega_{\text{eff}}^{\left(I\right)}|\leq \omega_{m}/2$, it is simple to keep track of the resonance conditions. In the special case $|\omega_{\text{eff}}^{\left(I\right)}|\approx |\omega_{\text{eff}}^{\left(S\right)}|\approx |\omega_{m}|/2$, the ZQ and DQ resonance conditions are fulfilled at the same time, implying that a single scaling factor is not sufficient for describing spin dynamics.

### Adiabatic transfers

An adiabatic polarization transfer corresponds to an inversion of the ZQ or DQ subspace. The relevant polarization operators in these subspaces are given by

$$I_{z}^{\text{ZQ}/\text{DQ}}=\frac{1}{2}(S_{z}\mp I_{z})$$ (22)

To adiabatically invert $I_{z}^{\text{ZQ}/\text{DQ}\to -I_{z}^{\text{ZQ}/\text{DQ}}}$, an adiabatic sweep is implemented by varying the offset (or resonance mismatch) in the ZQ/DQ subspace slowly from large positive values ($\Delta \omega_{\text{eff}}^{\mp }\gg 0$) through zero ($\Delta \omega_{\text{eff}}^{\mp }=0$) to large negative values ($\Delta \omega_{\text{eff}}^{\mp }\ll 0$). In general, the offset term in the ZQ/DQ subspace is not equivalent to the electron offset Ω*_S_*. For instance, in the adiabatic NOVEL DNP sequence, the offset in the ZQ/DQ subspace is determined by the mismatched Rabi field ω_1_(*t*). Figure 2B shows the schematic diagram of the described adiabatic sweep. The adiabatic sweep confers the additional advantage of avoiding cyclic behavior, i.e., backtransfer of nuclear polarization to the electron spin is excluded.

For a more quantitative treatment, we assume that (i) only one resonance condition is swept during the experiment, and (ii) the changes in the scaling factors and the electron spin component along the effective field are sufficiently slow relative to the change in the effective fields, i.e., an adiabatic process. The sweep approaches this idealized picture for large critical adiabaticity *Q*_crit_ at the moment the resonance condition is passed (*35*, *36*).

$$Q_{\text{crit}}^{\mp }=\frac{1}{4}\frac{{\left(Ba_{\mp }\right)}^{2}}{\frac{d}{\text{dt}}\Delta \omega_{\text{eff}}^{\mp }(t)}$$ (23)

With this critical adiabaticity, polarization-transfer efficiency can be computed by the Landau-Zener formula

$$\langle I_{z}\rangle =\pm \frac{\gamma_{e}}{\gamma_{n}}\langle \rho_{0}|{\tilde{S}}_{z}\rangle (1-\text{exp}\;(-\frac{\pi }{2}Q_{\text{crit}}^{\mp }))$$ (24)

Relaxation of the electron spin, which we neglected so far, imposes a lower limit on the sweep rate of ω_eff_ and thus an upper limit on critical adiabaticity. In practice, the compromise between lowering critical adiabaticity and accepting relaxation losses needs to be found experimentally for each DNP scheme and sample class. Nevertheless, as the scaling factor *a*_∓_ and the resonance conditions depend only on the waveform, good initial guesses and waveform parameter ranges can be estimated theoretically.

## MATERIALS AND METHODS

### Numerical calculation of effective fields and scaling factors

All numerical calculations were implemented in MATLAB (The MathWorks Inc.) and follow the treatment outlined in the Detailed theory section. An example script that calculates the effective fields and scaling factors can be found in the Supplementary Materials. First, the trajectory of the electron spin under a given irradiation scheme is calculated using quaternions, which directly yield the overall flip angle and the effective field. Then, the trajectory is transformed into a cyclic frame, where it is Fourier-transformed to generate the coefficients. One of the Fourier coefficients corresponds to the scaling factor *a*_±_. The same procedure can be applied to cases when an offset is present. MATLAB functions from EasySpin (*37*) were used for quaternion calculations.

### Spinach simulations

The numerical simulation of BEAM-DNP was performed using Spinach (*38*). A three-spin electron-proton-proton system was used, with a g-tensor of [2.0046 2.0038 2.0030], e-n distances of *r*_1_ = 4.5 Å and *r*_1_ = 6.5 Å, polar angles of θ_1_ = 0^∘^ and θ_2_ = 90^∘^, and azimuthal angles of ϕ_1_ = 0^∘^ and ϕ_2_ = 70^∘^. The relaxation effect was implemented via the Levitt-Di Bari approach (*39*) with *T*_1,e_ = 2.5 ms, *T*_2,e_ = 5 μs and *T*_1,n_ = 36 s, *T*_2,n_ = 1 ms. A two-angle Lebedev grid (*40*) with 194 orientations was used.

### Sample preparation

A 5 mM sample of OX063 trityl radical in DNP juice (glycerol-d_8_:D_2_O:H_2_O, 6:3:1 by volume) at 80 K was used for all experiments. In detail, 1.65 mg of trityl radical (molecular weight = 1359 g mol^−1^, 1.2 μmol) was dissolved in 24.3 μl of H_2_O and 72.9 μl of D_2_O. Of the resulting solution, 48.6 μl was then added to 72.9 μl of gly-*d*_8_. A volume of 40 ml of the final solution was transferred to a 3-mm–outer diameter quartz capillary and flash-frozen in liquid nitrogen before the measurements.

### Instrumentation and EPR/NMR spectroscopy

All experimental data were acquired on a home-built X-band spectrometer, which is based on the design described by Doll *et al.* (*41*). Notable differences compared to the earlier design are that a 1.8 GSamples/s digitizer (SP Devices ADQ412) was used and that the temperature of 80 K was achieved with a cryogen-free cryostat (Cryogenic Limited). Mw pulses were generated with an AWG model M8190A (Keysight) and amplified with a 1-kW traveling wave tube (TWT) amplifier (Applied Systems Engineering). A standard Bruker EN4118A-MD4 ENDOR resonator was used, with an external rf tuning and matching circuit. NMR experiments were performed using a Stelar PC-NMR spectrometer. An Arduino board was used to count TWT gate triggers of the EPR spectrometer, each corresponding to an *h* increment (Fig. 1). The Arduino board triggers the NMR acquisition after *h* loops.

Fourier Transform (FT) EPR spectra were acquired by a chirp echo sequence with linear chirp pulses spanning 300 MHz, a duration of 200 ns (π/2) and 100 ns (π) and an interpulse delay of 2 μs. All EPR and NMR signals were processed in MATLAB. All experimental results presented in this work were acquired within a single session; i.e., the sample was not moved between different DNP experiments.

### Pulse sequences and enhancements

The DNP pulse sequences used in this work are shown in Fig. 1. A train of ^1^H saturation pulses (eleven 100^∘^ pulses spaced by 1 ms) was applied in the beginning. Each DNP element was repeated *h* times, with a total buildup time *T*_DNP_ = *h* × *t*_rep_. The contact time *t*_contact_, during which the mw is turned on, is generally much shorter than the repetition time *t*_rep_, due to a 1% duty cycle limit of the TWT. The ^1^H NMR signal was then read out with a solid echo sequence composed of two 2.5 μs 90^∘^ pulses separated by a delay of *t*_SE_ = 80 μs. A conventional eight-step phase cycle was used with {*x*, *x*, *y*, *y*, − *x*, − *x*, − *y*, − *y*} for the first pulse and detection and {*y*, − *y*, *x*, − *x*, *y*, − *y*, *x*, − *x*} for the second pulse. The proton spectrum at thermal equilibrium was acquired using similar parameters except without mw irradiation, and a delay of 180 s ≈ 5 × *T*_1, n_ was used in between the 660 accumulated scans. The *T*_1,n_ = 36 s was determined both with a saturation recovery sequence and by the decay of polarization after DNP (see the Supplementary Materials).

For most cases, we report the polarization enhancement ε*_P_*, given by the ratio of the DNP-enhanced signal intensity divided by the signal intensity at thermal equilibrium. These values can be different from simple mw on/off signal enhancements recorded with the same delay, because the DNP buildup time *T*_B_ can be much shorter than *T*_1,n_. For most parameter optimizations, we used a repetition time *t*_rep_ of 1 ms and a buildup time *T*_DNP_ of 2 s. Buildup curves were acquired by changing the value of *h*, and with variable repetition times mentioned in the respective figures and tables.

## RESULTS

In this section, we apply the theory and design procedures outlined earlier for DNP experiments at X-band frequencies (9.5 GHz) using both low-power and high-power mw. The former involves variants of the recently published XiX-DNP experiments (*28*), while the latter involves development of a new pulse sequence, BEAM, with improved performance relative to previous NOVEL-DNP experiments (*16*, *17*, *19*).

### Low-power XiX-DNP

**Fig. 3.**
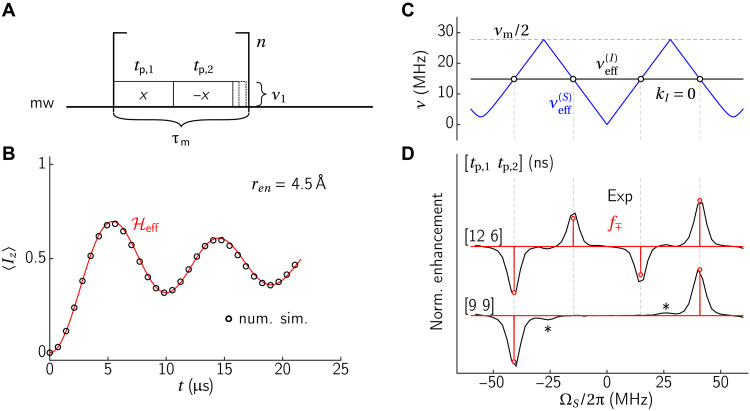
Numerical and experimental analysis of the XiX-DNP experiment. (**A**) Pulse sequence for the mw part of the DNP experiment. (**B**) Comparison of *S_z_* → *I_z_* polarization transfer efficiencies calculated using the effective Hamiltonian in Eq. 1 (red) with a full numerical simulation (black circles). A two-spin e-^1^H spin pair with a distance *r_en_* = 4.5 A° is used in the numerical simulations. (**C** and **D**) Resonance conditions and theoretical and experimental enhancements for XiX-DNP with ν_1_ = 4 MHz, *t*_contact_ = 8 μs τ_rep_ = 1 ms, *T*_DNP_ = 2 s as a function of the electron offset frequency. (C) The absolute value of the effective fields $\nu_{\text{eff}}^{\left(S\right)}$ (blue) and $\nu_{\text{eff}}^{\left(I\right)}$ (black) as a function of the mw offset, for *t*_p,1_ = *t*_p,2_*** =*** 9 ns. Resonance conditions are indicated as black circles. (D) Experimental enhancements for different combinations of *t*_p,1_ and *t*_p,2_ (given in brackets) with fixed total modulation period (black), and theoretical predictions based on Eq. 3, evaluated on the resonance conditions (red). Small additional peaks are due to higher-order processes involving two protons. The calculation in (B) was done at an electron offset of 40.89 MHz.

Figure 3A shows the mw part of the XiX-DNP pulse sequence consisting of two oppositely phased pulses repeated *n* times, leading to a total contact time of *t*_contact_ = *n* × τ_m_ = *n* × (*t*_p,1_ + *t*_p,2_). Assuming an mw field with an amplitude of ν_1_ = 4 MHz (we use ω for angular frequencies and ν = ω/2π for linear frequencies) and an offset slightly above 40 MHz, *t*_*p*,1_ = *t*_*p*,2_ = 9 ns, this leads to the calculated transfer profiles shown in Fig. 3B when using fully numerical simulations (black circles) or only the effective Hamiltonian in Eq. 1. This good agreement between the two curves confirms that the derived effective Hamiltonian is correct.

Next, Fig. 3C shows the electron and nuclear effective fields as well as the matching conditions as a function of the electron offset. In this example, $\nu_{\text{eff}}^{\left(I\right)}=\nu_{I}$ and *k_I_* = 0 for all resonance conditions. Since low-power mw irradiation is used, the electron effective field is mainly dominated by the electron offset. We note the reflection at ν_m_/2, which is a consequence of our particular choice of convention for the effective field.

When a low-power sequence is applied at frequency Ω*_S_*/2π and modulated with frequency ν_m_ = 1/τ_m_, one can treat it like a multifrequency irradiation with frequencies $\frac{\Omega_{S}}{2\pi }\pm m\times \nu_{m}$, where *m* is an integer number. For low-power irradiation and narrow EPR lines, only one of these components will lead to DNP, namely, the ones that hit the usual SE resonance condition Ω*_S_* ≈ ± ω*_I_*. While this approach breaks down if the nutation frequency ν_1_ becomes comparable to ν_m_, our general approach still holds.

Figure 3D shows the experimental results and calculated *f*_∓_ (Eq. 3, evaluated on the resonance conditions) for different combinations of *t*_p,1_ and *t*_p,2_ (but with a constant sum *t*_p,1_ + *t*_p,2_). The bottom case with *t*_p,1_ = *t*_p,2_ corresponds to the sequence introduced by Mathies *et al.* (*28*). Clearly, both the positions and the relative intensities of the matching conditions are well predicted. The small peaks visible in the experimental data correspond to a three-spin electron-^1^H−^1^H transition (see the Supplementary Materials). If both pulses have the same length (the bottom trace in Fig. 3D), the resonance condition at the usual SE offset (Ω*_S_*/2π ≈ *ν_I_*) is still fulfilled, but the scaling factor is zero. This is consistent with the multifrequency irradiation view, because the intensity at $\frac{\Omega_{S}}{2\pi }\pm 0\times \nu_{m}$ is zero if *t*_p,1_ = *t*_p,2_. In conclusion, this figure shows that the resonance conditions alone are not enough to characterize the DNP performance and that the theoretical scaling factors reliably predict the relative DNP enhancement.

**Fig. 4.**
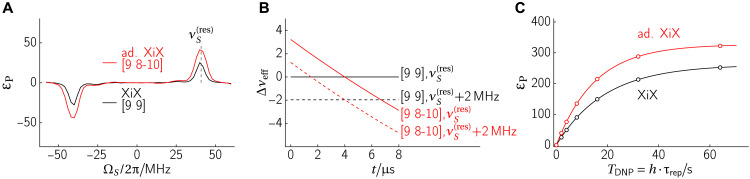
Experimental comparison of the diabatic (*t*_*p*,1_ = *t*_*p*,2_ = 9 ns, black) and adiabatic (*t*_*p*,1_ = 9 ns, *t*_*p*,2_ = 8–10 ns, red) XiX-DNP. (**A**) XiX-DNP mw offset profile with 2 s of buildup time. (**B**) Mismatch between nuclear and electron effective fields for XiX (black) and its adiabatic version (red) as a function of contact time. Solid lines show an exactly matched (Δν_eff_ = 0) resonance condition around 40 MHz, whereas the dashed line describes a scenario of a shifted resonance condition by 2 MHz. All lines except the black dashed line cross the Δν_eff_ = 0 line, and hence, DNP will take place. This clearly shows the mismatch compensating feature exhibited by adiabatic sequences. (**C**) Experimental ^1^H buildup curves with a repetition time of *t*_rep_ = 1 ms. XiX-DNP: ε_max_ = 261, *T*_B_ = 19.0 s; adiabatic XiX-DNP: ε_max_ = 327, *T*_B_ = 15.1 s.

The performance of the XiX-DNP experiment may be further improved by adiabatically sweeping the effective fields through the resonance condition, i.e., slowly increase *t*_p,2_ upon increasing the loop number *n* (Fig. 3A). The improved enhancement is demonstrated in DNP experiments (Fig. 4A), where the adiabatic version of XiX-DNP with the second pulse swept from 8 to 10 ns (red line) clearly outperforms its diabatic counterpart proposed by Mathies *et al.* (*28*) (black line). Figure 4B shows the time dependence of the effective field mismatch Δ*ν*_eff_(*t*) for the diabatic and adiabatic variants. The black lines correspond to the diabatic variant with fixed timing. Exactly at the offset of 40.89 MHz, the effective field mismatch is exactly zero (black solid line), and it does not change over time. Under these conditions, the transfer is optimal. However, if the offset is 2 MHz off—a reasonable value given that the full width at half maximum (FWHM) of trityl is ∼5 MHz—the effective fields are also mismatched by about 2 MHz (black dashed line). Consequently, the DNP matching condition is not fulfilled, and the DNP transfer is entirely quenched for a system with small hyperfine couplings. In contrast, it is evident that the effective field of the adiabatic variant (red) crosses zero in both cases, leading to polarization transfer for a broader distribution of electron offsets. Last, Fig. 4C shows the experimental buildup curve of the two XiX-DNP sequences, which clearly shows the advantages of implementing adiabatic XiX-DNP. A comparison of enhancement and buildup times of SE, XiX, and adiabatic XiX-DNP for two different repetition times is shown in Table 1, together with the results of the high-power sequences.

**Table 1.** Enhancements, ε_max_, buildup times, *T*_B_, and sensitivity per unit time (i.e., signal per square root of time), $\varepsilon_{\text{max}}\times \sqrt{T_{1,n}/T_{B}}$, for different DNP pulse sequences, optimized individually. RA-NOVEL and ad. BEAM were measured with an additional flip-back pulse after the DNP contact. ASE, adiabatic solid effect (*23*). *T*_1,n_ = 36.2 s. *T*_1,e_ = 2.5 ms.

		**SE**	**ASE**	**XiX**	**ad. XiX**	**NOVEL**	**RA-NOVEL**	**ad. BEAM**
*t*_rep_ = 1 ms	ε_max_	300	360	261	327			
	*T*_B_/s	15.9	11.9	19.0	15.1			
	$\varepsilon_{\text{max}}\cdot \sqrt{\frac{T_{1,n}}{T_{B}}}$	453	629	361	507			
*t*_rep_ = 2 ms	ε_max_	269	360	221	301	314	321	342
	*T*_B_/s	19.5	14.1	22.9	18.3	10.1	8.3	8.7
	$\varepsilon_{\text{max}}\cdot \sqrt{\frac{T_{1,n}}{T_{B}}}$	367	578	278	424	593	671	701
*t*_rep_ = 5 ms	ε_max_	190	309			297	335	361
	*T*_B_/s	25.4	18.3			14.9	12.1	12.1
	$\varepsilon_{\text{max}}\cdot \sqrt{\frac{T_{1,n}}{T_{B}}}$	227	435			463	580	626

### High-power BEAM-DNP

**Fig. 5.**
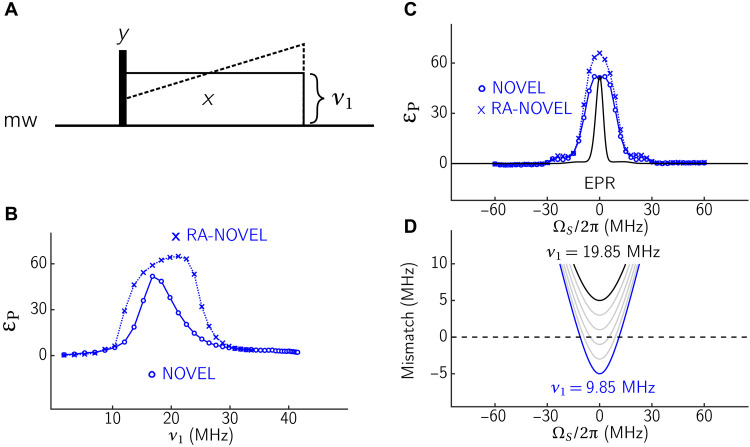
Results obtained with the NOVEL and RA-NOVEL sequences. (**A**) Pulse sequence for NOVEL and RA-NOVEL (dashed) DNP. (**B**) Experimental DNP enhancement as a function of the spin-lock strength (Rabi frequency) ν_1_ after 2 s of DNP (applied on resonance). Note that the mw inhomogeneity inside the resonator is quite pronounced, and the given value of ν_1_ is the maximum of the nutation spectrum. Off-resonance effects in the nutation experiment can lead to a slight overestimation of ν_1_. (**C**) DNP mw offset profiles for both NOVEL sequences (blue) and the EPR spectrum (black) with an arbitrary scale. Optimized power parameters determined from (B) were used. (**D**) Calculated resonance mismatch (Eq. 4) as a function of the offset for RA-NOVEL. The adiabatic sequence begins with a large negative Δν_eff_ ≪ 0 (blue), which slowly increases toward Δν_eff_ ∼ 0 (gray), and ends with a large positive Δν_eff_ ≫ 0 (black). DNP occurs whenever the lines cross Δν_eff_ = 0.

We will now analyze high-power pulsed DNP sequences. We start with NOVEL (*16*–*18*) and its adiabatic version, the RA-NOVEL-DNP (Fig. 5A) (*19*). We will then show how a simple amplitude modulation can be used to improve its bandwidth.

For NOVEL, the electron spin-lock strength has to match the nuclear Zeeman frequency, ν_1_ ≈ *ν_I_*, while for RA-NOVEL, the nutation frequency is slowly increased from below the matching condition to above it in a linear fashion. Although other amplitude modulation regimes were examined, no major improvement was observed at long contact times (*19*). Figure 5 (B and C) compares the DNP performances of the NOVEL sequences as a function of the (average) Rabi field *ν*_1_ and the offset Ω*_S_*/2π. The plots show that RA-NOVEL is more tolerant toward ν_1_ mismatch and hence leads to higher DNP enhancements. In addition, the calculated mismatch plot (Fig. 5D) also predicts that the adiabatic sequence can moderately improve the bandwidth for small couplings. Nevertheless, RA-NOVEL experiments did not show an improved offset compensation, most likely due to the more dominant mw Rabi field inhomogeneity (about 18%) across the sample.

**Fig. 6.**
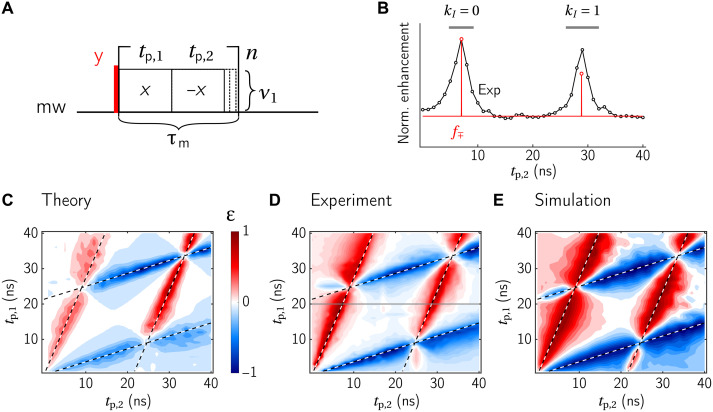
Resonance conditions of BEAM. (**A**) Pulse sequence of BEAM-DNP. (**B**) Experimental (black) and calculated (red) DNP performance as a function of *t*_p,2_ with fixed *t*_p,1_ = 20 ns. The ranges for the adiabatic sweeps are marked by gray bars. (**C**) Calculated relative enhancement (Eq. A11, based on the effective Hamiltonian) including offset distributions (5 MHz FWHM) and ν_1_ inhomogeneity (6 MHz FWHM centered at 32 MHz). (**D**) Experimental BEAM-DNP enhancement as a function of *t*_p,1_ and *t*_p,2_ with *T*_DNP_ = 1 s. The observed resonance conditions matches well with the theory (black and white dashed lines). The solid gray line indicates the position of (B). (**E**) Spinach simulation on a spin system described in Materials and Methods. The experiments were performed using on-resonance (Ω*_S_* = 0) mw irradiation and a Rabi field of ν_1_ ∼ 32 MHz, which is twice of that used for NOVEL. Other experimental details include *t*_contact_ = 8 μs, *t*_rep_ = 1 ms.

Motivated by these results and also our previous works in designing broadband solid-state NMR recoupling sequences (*42*), we hypothesized that a broadband pulsed DNP sequence can be designed by combining XiX and NOVEL, i.e., a BEAM-DNP (Fig. 6A). Similar to NOVEL (but unlike the XiX-DNP), BEAM-DNP is a spin-locked experiment. In addition, the sequence can be made adiabatic by slowly varying *t*_p,2_ through one of the matching conditions, which are shown as dashed lines in the two-dimensional plot of a theoretical prediction using the Hamiltonian in Eq. 1 (Fig. 6C, see also Eq. 16). This calculation included distributions of electron offsets and Rabi fields. The intensity and width of the resonance conditions already hint at the robustness of them with respect to these parameters.

Note that in the particular case of on-resonance BEAM, there is again a conceptually simple way of determining the resonance condition. On such a condition, the effective fields are the same. From this, it follows that also the effective flip angles have to match (up to a multiple of 2π). In BEAM, the effective flip angle of the nuclear spin is given by $\beta_{\text{eff}}^{\left(I\right)}=\omega_{I}(t_{p,1}+t_{p,2})$, while the effective flip angle of the electron spin is given by $\beta_{\text{eff}}^{\left(S\right)}=\omega_{1}(t_{p,1}-t_{p,2})$. Thus, the resonance conditions in Fig. 6 (C to E) follow the equation ω*_I_*(*t*_p,1_ + *t*_p,2_) = ω_1_(*t*_p,1_ − *t*_p,2_) + *m* · 2π, where *m* is again an integer. While such analytical treatments are possible for simple sequences performed with ideal parameters, it becomes nontrivial when offsets are present or when more complicated modulation schemes are used.

Figure 6B shows the DNP enhancement as a function of *t*_p,2_ with a fixed value of *t*_p,1_ = 20 ns. One can see that the position of the resonance conditions is well predicted, and that the calculated *f*_∓_ (Eq. 3) matches the relative DNP performance well, despite the fact that the Rabi field inhomogeneity and mw offsets were simply neglected in these calculations. We have labeled the two different resonance conditions (*k_I_* = 0 and *k_I_* = 1) for later reference (vide infra), and the sweep range of the adiabatic variants are indicated by gray bars. The experimental enhancement as a function of both pulse lengths is shown in Fig. 6D. Again, the positions of the resonance conditions are well predicted by the theory. There are some differences in the width and intensity that are expected from the simplistic two-spin model we are using. In addition, numerical results (Fig. 6E) using Spinach (*38*) shows that our theory reliably predicts the resonance conditions for BEAM-DNP. While numerical simulations of small spin systems can include more details, such as electronic relaxation, they are still not encompassing all the details in the complete DNP process. In this case, our (semi-)analytical theory is very helpful in planning and setting up preliminary experiments.

**Fig. 7.**
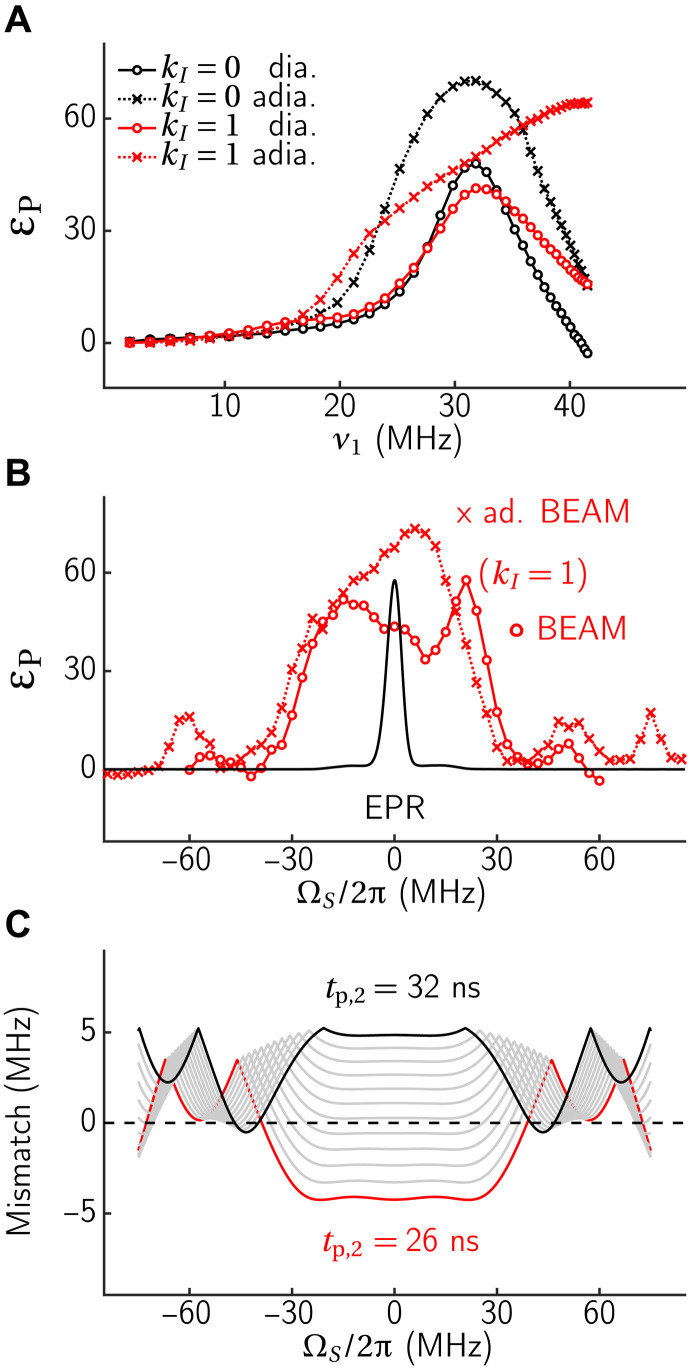
Robustness of the BEAM experiment. Experimental BEAM-DNP enhancement as a function of (**A**) the Rabi field ν_1_ for the diabatic and adiabatic versions of the respective resonance conditions and (**B**) offset Ω*_S_*/2π, and an EPR spectrum is included here for reference (black). The BEAM parameters were *t*_p,1_ = 20 ns, *t*_contact_ = 8 μs, τ_rep_ = 1 ms, *T*_DNP_ = 2 s. The pulse length of the second pulse, *t*_p,2_ was fixed in the case of (diabatic) BEAM (“∘”) to 7 ns (*k_I_* = 0) and 29 ns (*k_I_* = 1), while it was swept over 4.75 to 9.25 ns (*k_I_* = 0) or 26–32 ns (*k_I_* = 1) for adiabatic BEAM (“x”). (**C**) Calculated offset-dependent mismatch for the *k_I_* = 1 condition. The adiabatic sweep begins with *t*_p,2_ ***=*** 26 ns (red), through the intermediate stages (gray), and ends at *t*_p,2_ ***=*** 32 ns (black).

The BEAM-DNP enhancement as a function of ν_1_ is shown in Fig. 7A, and it is evident that the adiabatic BEAM outperforms its diabatic counterpart. For one of the resonance conditions, the best transfer was achieved with the highest power available. A closer inspection at the *k_I_* = 0 and *k_I_* = 1 resonance conditions reveals that the latter condition is more robust with respect to the spin-lock field strength, in agreement with the experimental data (Fig. 7A). Figure 7B shows the BEAM DNP frequency profile (constant *B*_0_ field with varying mw center frequency) for the *k_I_* = 1 case. A maximum mw power was used for the π/2 pulse for a maximum bandwidth, but the spin-lock field was adjusted at each offset position according to the mw resonator (see the Supplementary Materials). Note that the mw power adjustment was not possible for the adiabatic BEAM due to the limited mw power available. The small enhancements at larger offsets ($\frac{\Omega_{S}}{2\pi }=\pm 60$ MHz) are due to the matched resonance conditions during the adiabatic sweep, where the offset-dependent mismatch during the contact period is explicitly calculated (Fig. 7C).

As discussed in the analysis performed for RA-NOVEL, DNP occurs when the mismatch is zero (diabatic case) or passes zero (adiabatic case). The theory shows that the mismatch for BEAM DNP is quite offset-tolerant, as visible in Fig. 7C. In other words, the mismatch hardly varies by more than 1 MHz over the ∣Ω*_s_*/2π∣ < 30 MHz range. Hence, the theory implies that BEAM will be a broadband sequence, and this was verified experimentally.

**Fig. 8.**
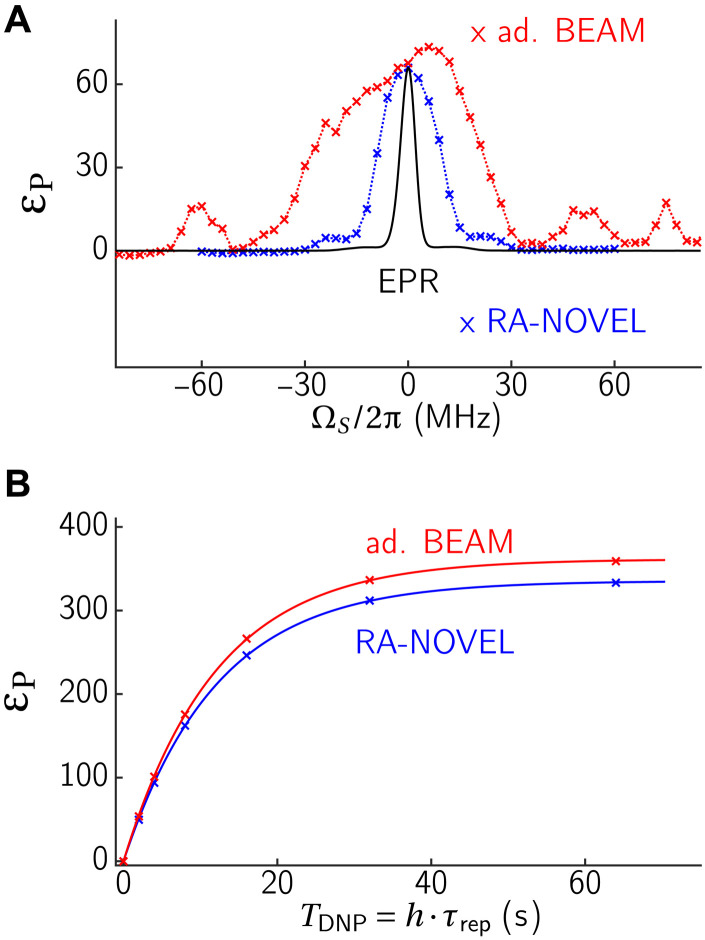
Comparison of RA-NOVEL and BEAM. (**A**) DNP frequency profile of RA-NOVEL and adiabatic BEAM (*k_I_* = 1) DNP with 2 s buildup time. (**B**) DNP buildup curve using a repetition time of 5 ms. The buildup curves were fitted with exponential functions ε_P_(*T*_DNP_) = ε_max_(1 − exp (−*T*_DNP_/*T*_B_)). The buildup curves were measured using different repetition times τ_rep_ = 1 to 20 ms, where *t*_rep_ = 5 ms yielded the largest ε, and *t*_rep_ ***~*** 2 ms resulted in the highest $\varepsilon_{\text{max}}/\sqrt{T_{1,n}/T_{B}}$. Ninety-degree flip-back pulses were applied after each DNP contact to replenish the electron Zeeman spin bath. Exact BEAM parameters are given in Fig. 7.

It is evident that the increased Rabi field ν_1_ used in BEAM has resulted in a ∼3× higher bandwidth compared to RA-NOVEL (Fig. 8).

Moreover, we also measured the buildup curves (Fig. 8B), and the results are summarized in Table 1. In summary, adiabatic BEAM has outperformed RA-NOVEL with a higher ε_max_ of ≈ 361 and $\varepsilon_{\text{max}}\cdot \sqrt{T_{1,n}/T_{B}}\approx$ 701, compared to ε_max_ of ≈335 and $\varepsilon_{\text{max}}\cdot \sqrt{T_{1,n}/T_{B}}\approx$ 671 for RA-NOVEL. Although the improvement of adiabatic BEAM over RA-NOVEL is marginal (≤8%), a larger relative gain can be envisaged when applied to other more generic DNP polarizing agents, which usually have broader lines than OX063, and hence, offset compensation becomes critical. Table 1 also shows a comparison with NOVEL and the low-power sequences mentioned above. Note that RA-NOVEL and BEAM are efficient enough that they are mostly limited by *T*_1, e_ and the spin diffusion rate between the nuclear spins. The low-power sequences can thus partially compensate for the lower efficiency by using a faster repetition rate. This would not be possible for faster relaxing electron spins or in single-contact DNP experiments.

## DISCUSSION

We have demonstrated a general theoretical treatment applicable to periodic mw DNP sequences in static samples. This is realized by analyzing the resonance conditions and determining the scaling factors, which encode the details in a sequence—Rabi fields, phases, amplitudes, and mw offsets. We showed here an example of how theory can help to improve existing sequences, such as XiX-DNP, and an example of how to design a broadband sequence that is robust against mw offsets. We show that adiabatic BEAM has outperformed RA-NOVEL by obtaining ε_max_ of ≈ 361 and $\varepsilon_{\text{max}}\cdot \sqrt{T_{1,n}/T_{B}}\approx$ 701 on trityl radicals at a temperature of 80 K, a field of 0.35 T, and under static conditions. While the adiabatic solid effect can achieve similar maximal enhancements of 360 (*23*), it has a slightly longer buildup time *T*_B_ (and thus a lower sensitivity enhancement of $\varepsilon_{\text{max}}\cdot \sqrt{T_{1,n}/T_{B}}\approx$ 629). The ASE is mostly suitable for narrow-line radicals, because it is inherently limited to a bandwidth equal to or lower than the nuclear Larmor frequency. Accordingly, we expect BEAM to perform better than the other sequences discussed in this work for radicals with broader EPR spectra.

Our theory implies that adiabatic BEAM (and the other sequences used in this work) only shows field-independent performance if the modulation frequency of the sequence and the mw Rabi fields are also scaled linearly with the external magnetic fields. As the mw power requirement for adiabatic BEAM performed here requires that the electron Rabi frequency is at least twice as large as the ^1^H Larmor frequency, it will be challenging to exploit it for high-field (>5 T) DNP NMR applications. Nevertheless, it could be satisfactorily fulfilled for polarizing ^13^C in diamond nitrogen-vacancy (NV) centers. For example, PulsePol, which requires an order of magnitude more mw power than adiabatic BEAM, was demonstrated to polarize ^13^C nuclei in diamond NVs at ∼0.17 T (*26*). In such situations, a broadband sequence that is robust against mw power inhomogeneity and offsets could be advantageous for quantum computing applications (*43*).

Last, we emphasize that our generalized theoretical framework is applicable not only to the design and understanding of pulsed DNP experiments but also in a much broader range of magnetic resonance, e.g., liquid- and solid-state NMR (*29*, *30*), pulsed EPR experiments using matching conditions (*13*, *44*), and possibly pulsed MAS-DNP experiments in the future.

## Supplementary Materials

### This PDF file includes:

Sections S1 to S7 Figs. S1 to S11
