# Supplementary material for: Designing broadband pulsed dynamic nuclear polarization sequences in static solids
Source: Sci Adv. 2022 Jul 15;8(28):eabq0536. doi: 10.1126/sciadv.abq0536 (PMC9286509; doi:10.1126/sciadv.abq0536)
Supplement: Supplementary file 1 — Sections S1 to S7 Figs. S1 to S11 [file sciadv.abq0536_sm.pdf]

Science Advances
AAAS

Supplementary Materials for

Designing broadband pulsed dynamic nuclear polarization sequences in static solids

Nino Wili *et al.*

Corresponding author: Nino Wili, nino.wili@alumni.ethz.ch; Kong Ooi Tan, kong-ooi.tan@ens.psl.eu

Sci. Adv. **8**, eabq0536 (2022)
DOI: 10.1126/sciadv.abq0536

This PDF file includes:

Sections S1 to S7
Figs. S1 to S11

S.1 Illustration of the interaction frame transformation

A central element in calculating the scaling factors $a_{\mp}$ is the interaction frame transformation with respect to the irradiation sequence and the transformation to a cyclic frame. Here, we give a concrete example of the elements of $R^{(\text{control})}$ and $R^{(\text{eff})}$ and their relationship. We consider an XiX-DNP experiment (parameters here not chosen to represent any good DNP sequence, but for easier explanation) with $\nu_1 = 4\,\text{MHz}$, $t_{\text{p},1} = 14\,\text{ns}$, $t_{\text{p},2} = 28\,\text{ns}$, and $\Omega_S/2\pi = 25\,\text{MHz}$ Figure S1**(a)** shows the elements of the initial interaction frame transformation $R^{(\text{control})}(t)$ plotted over one period τ_m. The blue curves denote the trajectory of the normal rotating frame operator S_z. Note that $R^{(\text{control})}(0) = \mathbb{1} \neq R^{(\text{control})}(\tau_\text{m})$, i.e. the trajectory is not cyclic with τ_m. This prohibits the straight-forward application of average Hamiltonian theory and is the reason for the subsequent transformations. Figure S1**(b)** shows the three-dimensional trajectory of the original S_z operator in the initial interaction frame (the three blue components in panel **(a)**). The trajectory of the first modulation period is marked in red. The end points of the subsequent five periods are shown as black dots in panel **(b)**. The overall rotation from one period to the next can be described by an effective field shown in gray. This can be understood as a constant effective field, which can be removed by flipping the frame such that the effective field is along z, and then going into an interaction frame with said effective field. The result of this transformation, $R^{(\text{eff})}$, is shown in Figure S1**(c)** and **(d)**. The z-axis in this new frame points along the effective field in Figure S1**(b)**, and the effect of the overall rotation was eliminated by a counter rotation, i.e. start and end points of the trajectories are now the same. In Figure S1**(c)**, all the coefficients are cyclic with time τ_m. A Fourier transform of the respective time-dependent coefficients directly yields the coefficients $a_{\chi z}^{(k)}$. An example script to perform these calculations in MATLAB is given below.

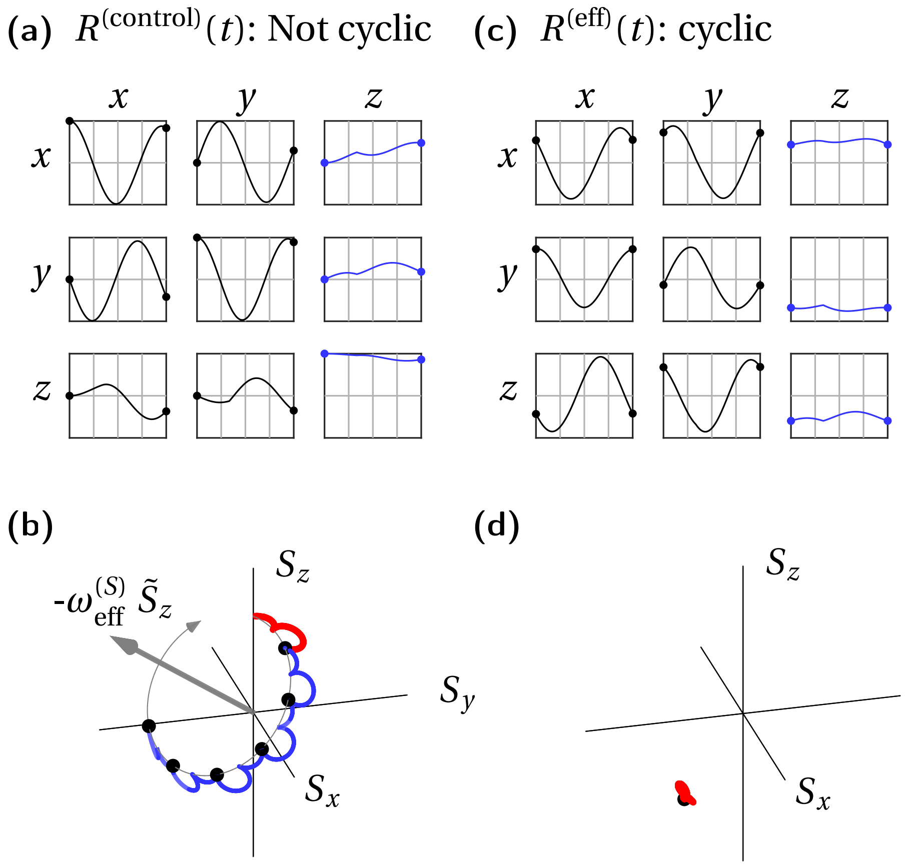

Fig. S1: **Illustration for the interaction frames employed in this work, on the example of XiX-DNP** (details in main text). **(a)** Illustration of the initial interaction frame transformation $R^{(\mathrm{control})}(t)$. The individual plots show the evolution of rotation matrix elements over one period τ_{m}. **(b)** Three-dimensional trajectory of the original S_z operator in the initial interaction frame (the three blue components in **(a)**). The trajectory of the first modulation period is marked in red. The end points of the subsequent five periods are shown as black dots. The effective field describes the overall rotation of the sequence. **(c)** and **(d)** The same trajectory as in **(a)** and **(b)**, but in the flipped, effective (or cyclic) frame. The start and end points are the same.

S.2 Calculation of Fourier coefficients and scaling factors for BEAM

In this section, we show an example of how to calculate the effective fields and scaling factors on the example of BEAM. Although BEAM is best applied on-resonant, we include here an offset of 5 MHz for illustration. The example can be found in the file `DNPexample_BEAM.m`.

S.2.1 Build the rf-irradiation

```
clear, close all

% add helper functions
addpath(genpath('./core/'))


%% define sequence parameters
nu_I=-14.83;              % Nuclear Zeeman frequency
nu_1=32;                    % Electron Rabi freq
tp1=20*1e-3;              % first pulse length in BEAM
tp2=(1+tp1*(nu_1-abs(nu_I)))/(abs(nu_I)+nu_1);            % second pulse length in BEAM


nu1_vec = nu_1*[1 1];
tp_vec = [tp1 tp2];
phi_vec = [0 180]/180*pi; % phases of the pulses
dt =0.1e-4;               % time step of numerical IFT

offset = 5;

rho0_vec = [-1 0 0]';

%% build rf
[ rf, time ]=build_rf(tp_vec,nu1_vec,phi_vec,dt);

%plotting
h = figure(1);
clf
hold on
plot(time*1e3,real(rf),'b')
plot(time*1e3,imag(rf),'r')
xlabel('t / ns')
ylabel('\nu_1 / MHz')
axis([-1 60 -40 40])
```

```
legend('real(rf)','imag(rf)','location','northeast')
```

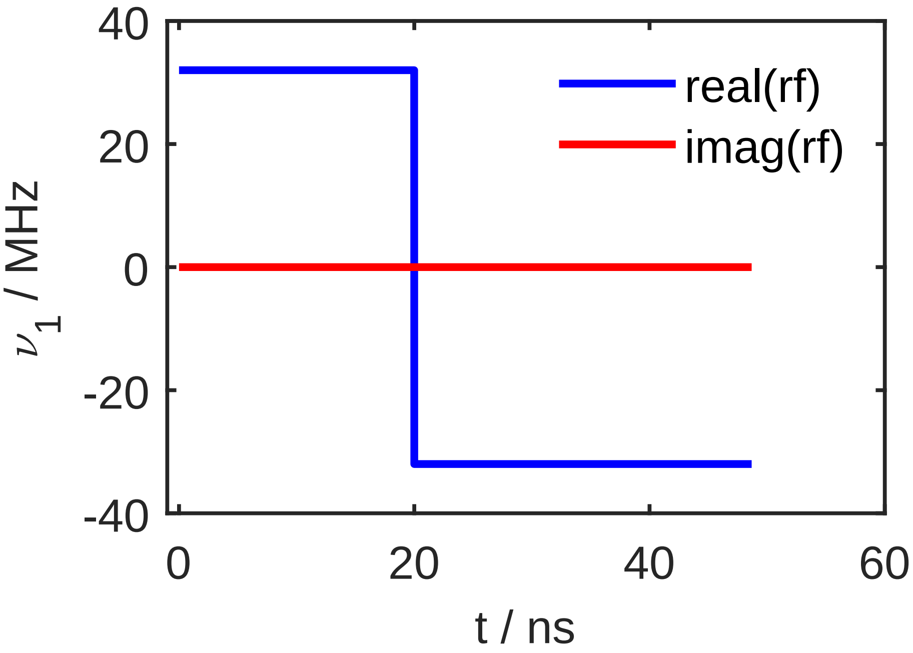

Fig. S2: **rf-irradiation scheme for the BEAM simulation.**

S.2.2 Calculate $R^{(\mathbf{control})}(t)$

```
% generate a vector of offsets the same length as rf
offset=offset*ones(size(rf));

%calculate modulation frequency
wmod=2*pi/(time(end)+dt);
nu_m = wmod/2/pi;


% pre-allocate rotation matrices
R_control = zeros(3,3,numel(time));
R_flipped = zeros(3,3,numel(time));


% build array of rotation quaternions for every step
q_pulse = zeros(4,numel(time));
for it = 1:numel(time)
    q_pulse(:,it)=quat_rf(abs(rf(it)),angle(rf(it)),offset(it),dt,'frame') ;
end


% multiply the quaternionas step-by-step and
% express them as an array of rotation matrices (for plotting only at this stage)
qtot = [1 0 0 0]';
q_control = zeros(4,numel(time));
for it=1:numel(time)
    q_control(:,it)=qtot;
    R_control(:,:,it) = quat2rotmat(qtot);
    qtot = quatmult(qtot,q_pulse(:,it));
end



% Plot IF in rotating frame
IF_plot(time,R_control,2);
```

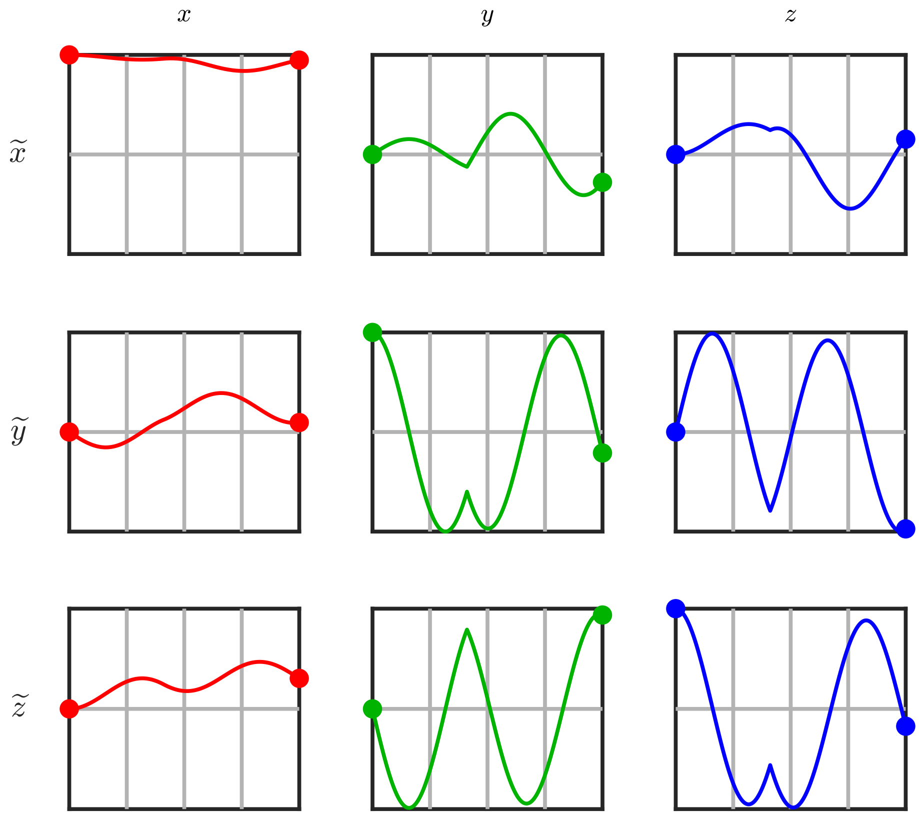

Fig. S3: **Time-dependence of each element of $R^{(\mathbf{control})}(t)$.**

S.2.3 Remove the effective field from $R^{(\text{control})}(t)$, determine $R^{(\text{eff})}(t)$

```
%% flip each quaternion of q_control (or R_control)
% such that the effective field is now along the z-axis

%determine the effective field (effective flip angle and direction)
[z_eff,beta_eff] = quat2eff(qtot);

% determine R_flip, or the corresponding quaternion, respectively
% see https://doi.org/10.1063/1.5123046 for details
u_z = [0 0 1]';
q_flip = cross(z_eff,u_z);
qr_flip = 1+u_z'*z_eff;
q_flip = [qr_flip; q_flip ];
q_flip = q_flip/sqrt(sum(q_flip'*q_flip));

% flip all quaternions/rot-matrices
q_flipped= zeros(4,numel(time));
for it=1:numel(time)
    q_flipped(:,it)=quatmult(q_flip,q_control(:,it));
    R_flipped(:,:,it) = quat2rotmat(q_flipped(:,it));
end

%% remove the effective field from the IFT, i.e. calculate R_eff(t)
% determine the effective field frequency
w_eff = beta_eff/(time(end)+dt);
nu_eff = w_eff/(2*pi);

%pre-allocate rotation quaternions/matrices
q_eff = zeros(4,numel(time));
R_eff = zeros(3,3,numel(time));

% calculate R_eff(t)
for it=1:numel(time)

    %calculate Rz(-weff*t),
    beta = -w_eff*time(it);
    q_z = [cos(beta/2) sin(beta/2)*[0 0 1]]';

    q_eff(:,it)=quatmult(q_z,q_flipped(:,it));
    R_eff(:,:,it) = quat2rotmat(q_eff(:,it));
end

```

```
IF_plot(time,R_eff,3);
```

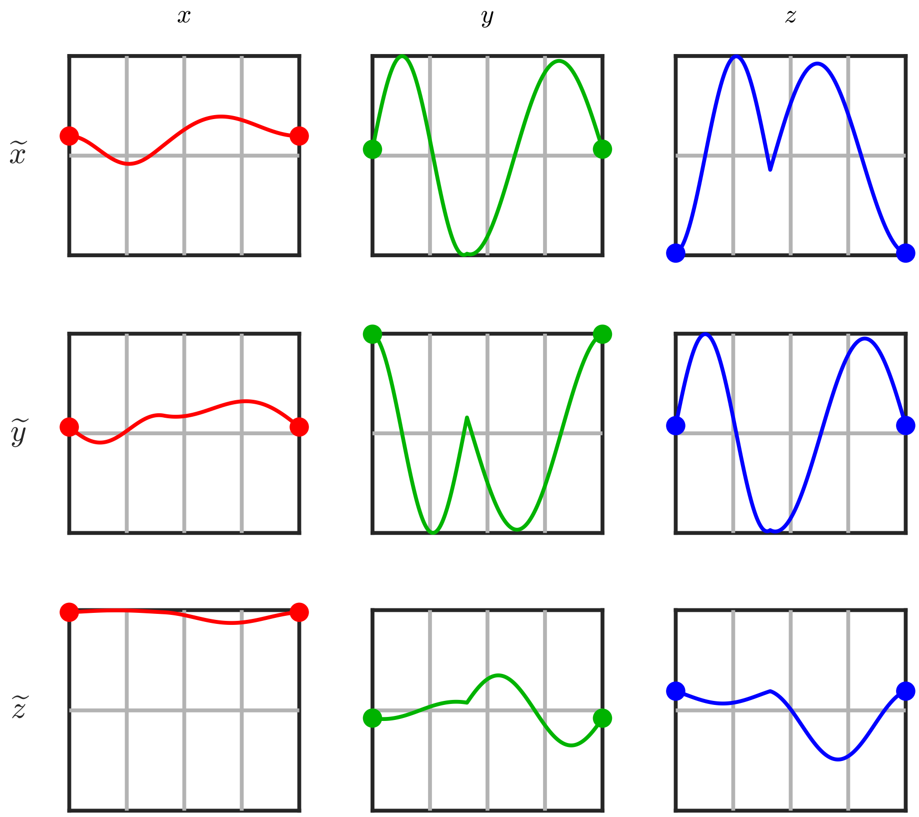

Fig. S4: **Time-dependence of each element of $R^{(\mathrm{eff})}(t)$.** Note that all elements are preiodic.

S.2.4 Calculate the Fourier coefficients and scaling factors

```
%%  calculate Fourier coefficients a^(k)
N=numel(time);
k_vec = ((0:N-1)-fix(N/2));
if mod(N,2)==0
    k_vec = k_vec(2:end);
end

A_k = zeros(3,3,numel(k_vec));


% Fourier transform each element (xx,xy,xz,...zz) of R_eff(t)
for ii=1:3
    for jj=1:3
        y = squeeze(R_eff(ii,jj,:));
        a = fftshift(fft(y))/numel(y);
        if mod(N,2)==0
            A_k(ii,jj,:)=a(2:end);
        else
            A_k(ii,jj,:)=a(1:end);
        end
    end
end


%extract the original z operator (should no oszillate during free
   evolution)
Az_k = squeeze(A_k(:,3,:));

%% extract the relevant scaling factor

[nu_eff_I,kI] = get_nu_eff_I(nu_I,nu_m);

kmax=max(k_vec);

ax = squeeze(Az_k(1,:));
ay = squeeze(Az_k(2,:));
ap = ax-1i*ay;
am = ax+1i*ay;
az = squeeze(Az_k(3,:));
```

```
k0 = kmax+1;
k = kmax+1+kI;
mk = kmax+1-kI;

rel_sign = sign(nu_eff*nu_eff_I);
if rel_sign == 1
    a_eff= -sqrt(ap(mk)*am(k)) ;
else
    a_eff= sqrt(am(mk)*ap(k)) ;
end

proj = z_eff'*rho0_vec;

f_pm = a_eff*proj
```

S.2.5 Compare with fully numerical calculation

```
%% comparison with numerical simulation

Ntheta = 31; %number of orientations

r=4.5; % e-n distance in Angstrom
Nrounds = 200; %number of repetitions of the DNP element

%caluclate the transfer numerically
[t_num,sig_num]=DNP_numerical(tp_vec,phi_vec,nu1_vec,offset,nu_I,rho0_vec,r,Ntheta,Nrounds);

h4=figure(4);
clf
hold on
plot(t_num,sig_num)

%% analytical calculation
%calculate the mismatch
delta_nu_eff = abs(nu_eff_piecewise(tp_vec,phi_vec,nu1_vec,offset))-abs(nu_eff_I);

%calculate the anisotropy of the hf-coupling
natural_constants
T = mu0/(4*pi)*gfree*bmagn*g1H*nmagn*1/(r*1e-10)^3/planck/1e6;

%generate orientations and corresponding weights
```

```
theta_vec = linspace(0,pi/2,Ntheta);
weights=sin(theta_vec); weights=weights/sum(weights);

%preallocate results
t_theo = t_num(1:10:end);
sig_theo = zeros(1,numel(t_theo));

%loop over orientations, sum up results
for itheta = 1:Ntheta

    theta=theta_vec(itheta);
    B=3*sin(theta)*cos(theta)*T;
    nu_pm = sqrt(B^2*a_eff^2/4+delta_nu_eff^2); %transfer frequency
    transfer_amp = a_eff^2*B^2/(4*nu_pm^2); %amplitude of transfer

    sig_theo = sig_theo+weights(itheta)*sign(a_eff)*proj*transfer_amp*...
                sin(1/2*(2*pi*nu_pm)*t_theo).^2;
end
plot(t_theo,sig_theo,'o')

axis([xlim -0.1 0.8])
xlabel('t / \mus')
ylabel('<I_z>')
legend('num.','theo.','location','northeast')
```

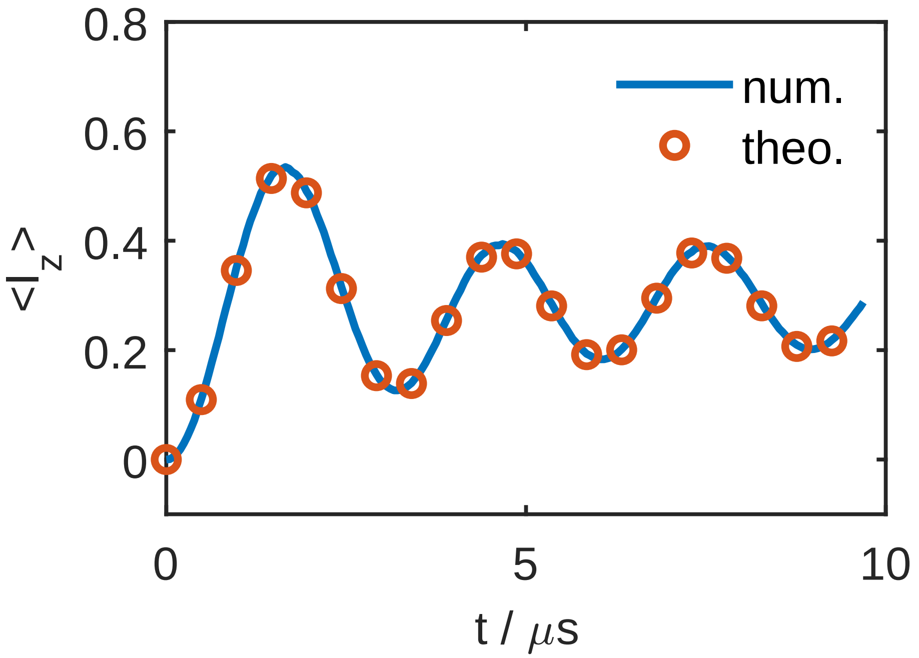

Fig. S5: **Fully numerical simulation vs. effective Hamiltonian calculation.**

S.3 Comparison of numerical and analytical computations for strongly coupled protons

Figure 3(b) in the main text showed a comparison of numerical and analytical computations for a proton at a distance of r_{en} = 4.5 Å. This is is already quite close. Here we show what happens if one looks at protons that are even closer (2.5 Å), see Figure S6. In this case, the hyperfine coupling is very strong. Nevertheless, the full first-order Hamiltonian (blue line) still perfectly describes the transfer. However, it is clear that only looking at the effective fields and the flip-flop terms (red) becomes more and more problematic for strong hyperfine couplings.

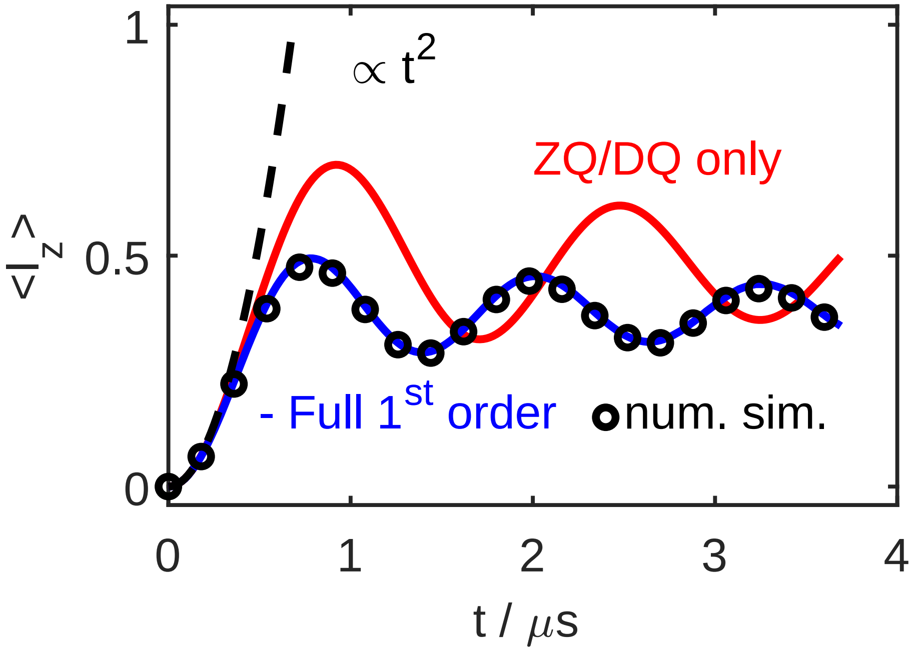

Fig. S6: **Influence of strong proton hyperfine couplings.** Comparison of $S_z \rightarrow I_z$ polarization transfer efficiencies calculated using an effective Hamiltonian including all first order terms (blue) or only the flip-flop terms (red) with a full numerical simulation (black circles). A two-spin e-^{1}H spin pair with a distance r_{en} = 2.5 Å is used in the numerical simulations. This is a very strongly coupled proton. The dashed line illustrates the initial build-up proportional to t^2.

S.4 Three-spin transitions

Some small features in the Experimental DNP profiles cannot be explained by the electron-nuclear two-spin model. It is well known that there are also electron-nuclear-nuclear three-spin transitions. A comprehensive treatment for them would require second-order average Hamiltonian theory. However, the position of these features can be estimated by simply doubling the nuclear Zeeman frequency and then looking for matching conditions in exactly the same way as in the two-spin case. This is shown in Fig. S7.

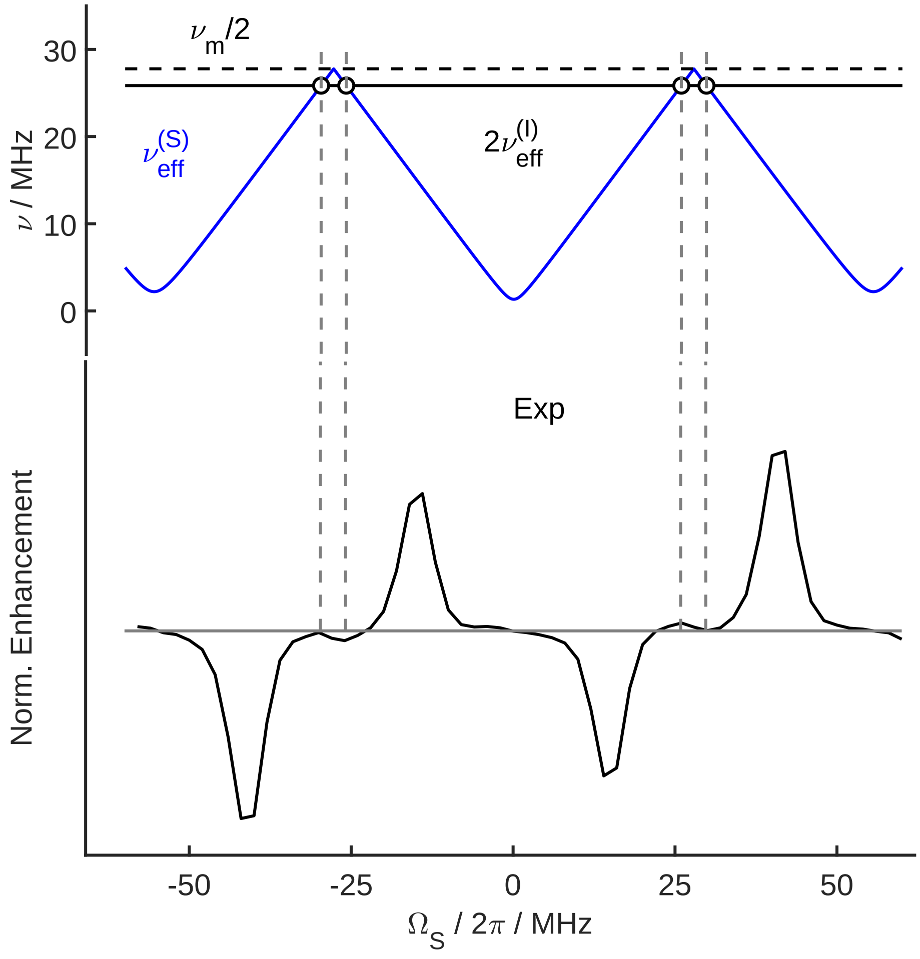

Fig. S7: **Predicted offsets for three-spin transitions for XiX DNP with $t_{p,1}$=12 ns and $t_{p,2}$=6 ns.**

S.5 Resonator profile

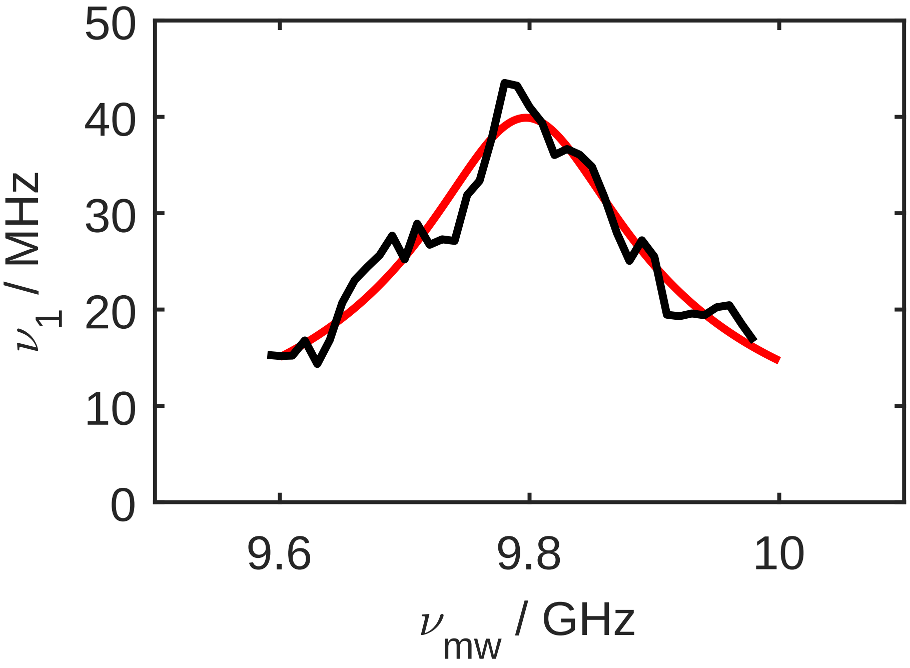

Fig. S8: **Resonator characterization.** Experimental resonator profile (black), determined by nutation experiments, and Lorentzian fit (red). $\nu_{1,max}$=40 MHz, Q=61.

S.6 Experimental determination of T_1, e

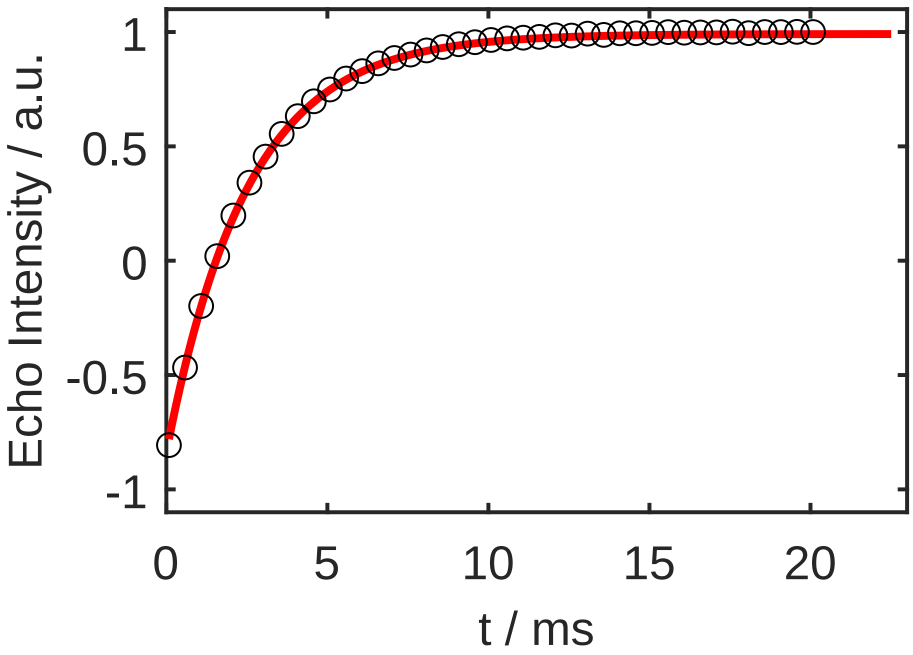

Fig. S9: **Inversion recovery data for the electron spin (black circles) and exponential fit (red).** $T_{1,e}$=2.5 ms
.

S.7 Experimental determination of T_1,n

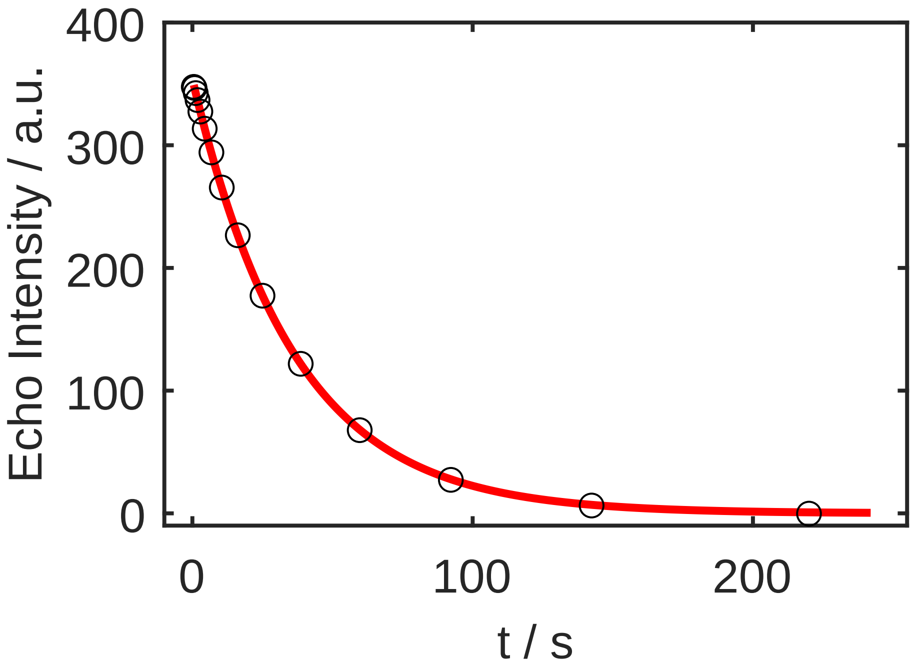

Fig. S10: **Experimental proton polarization decay after 60 s of adiabatic solid effect DNP (black circles) and exponential fit (red).** $T_{1,n}$=36.2 s

.

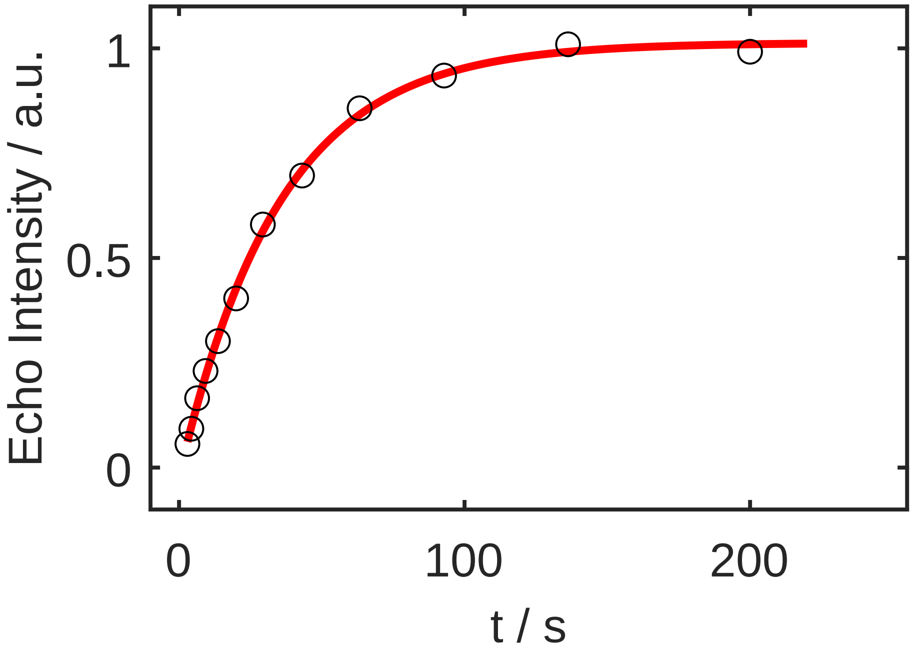

Fig. S11: **Proton saturation recovery data without DNP (black circles) and exponential fit (red).** $T_{1,n}$=35 s

.
